# Inverted chimeric RNAi molecules synergistically cotarget MYC and KRAS in KRAS-driven cancers

**DOI:** 10.1172/JCI187204

**Published:** 2025-07-22

**Authors:** Yogitha S. Chareddy, Hayden P. Huggins, Snehasudha S. Sahoo, Lyla J. Stanland, Christina Gutierrez-Ford, Kristina M. Whately, Lincy Edatt, Salma H. Azam, Matthew C. Fleming, Jonah Im, Alessandro Porrello, Imani Simmons, Jillian L. Perry, Albert A. Bowers, Martin Egli, Chad V. Pecot

**Affiliations:** 1 Curriculum in Genetics and Molecular Biology and; 2 Lineberger Comprehensive Cancer Center, and; 3 RNA Discovery Center University of North Carolina at Chapel Hill, Chapel Hill, North Carolina, USA.; 4 EnFuego Therapeutics Inc., Morrisville, North Carolina, USA.; 5 Division of Chemical Biology and Medicinal Chemistry, Eshelman School of Pharmacy;; 6 Center for Integrative Chemical Biology and Drug Discovery, Eshelman School of Pharmacy;; 7 Division of Pharmacoengineering and Molecular Pharmaceutics, Eshelman School of Pharmacy;; 8 Center for Nanotechnology in Drug Delivery; and; 9 Department of Chemistry; University of North Carolina at Chapel Hill, Chapel Hill, North Carolina, USA.; 10 Department of Biochemistry, School of Medicine, Vanderbilt University, Nashville, Tennessee, USA.; 11 Division of Oncology and; 12 Department of Medicine, University of North Carolina at Chapel Hill, Chapel Hill, North Carolina, USA.

**Keywords:** Oncology, Therapeutics, Drug therapy, Lung cancer, Oncogenes

## Abstract

Mutant KRAS has been implicated in driving a quarter of all cancer types. Although inhibition of the KRAS^G12C^ mutant protein has shown clinical promise, there is still a need for therapies that overcome resistance and target non-KRAS^G12C^ mutations. KRAS activates downstream MYC, which is also a difficult-to-drug oncoprotein. We have developed an “inverted” RNAi molecule with the passenger strand of a MYC-targeting siRNA fused to the guide strand of a KRAS-targeting siRNA. The chimeric molecule simultaneously inhibits KRAS and MYC, showing marked improvements in efficacy beyond the individual siRNA components. This effect is mediated by 5′-dT overhangs following endosomal metabolism. The synergistic RNAi activity led to a more than 10- to 40-fold improvement in inhibition of cancer viability in vitro. When conjugated to an EGFR-targeting ligand, the chimeric siRNA was delivered to and internalized by tumor cells. As compared with individual targeting siRNAs, the chimeric design resulted in considerably improved metabolic stability in tumors, enhanced silencing of both oncogenes, and reduced tumor progression in multiple cancer models. This inverted chimeric design establishes proof of concept for ligand-directed, dual silencing of KRAS and MYC in cancer and constitutes an innovative molecular strategy for cotargeting any two genes of interest, which has broad implications.

## Abstract 2

This study presents a novel chimeric siRNA that simultaneously targets KRAS and MYC, enhancing cancer inhibition in vitro and reducing tumor burden in vivo.

## Introduction

Mutations in the GTPase KRAS are responsible for driving nearly 25% of all cancers (1, 2). Normally involved in cell proliferation and differentiation, the *KRAS* proto-oncogene is often mutated at amino acid positions 12 and 13, which constitutively activates the KRAS protein by locking it into the active GTP-bound state (3). Until recently, KRAS was largely considered an “undruggable” target because the surface topology of the protein did not present a binding interface for a traditional small-molecule inhibitor (4, 5). However, studies have shown that the KRAS^G12C^ mutant form can create a stable nucleophilic binding pocket that can be targeted with covalent small-molecule inhibitors (6). This has led to the rapid development of clinical-stage KRAS^G12C^ inhibitors (7–9), including the approved drugs sotorasib and adagrasib. Although the KRAS^G12C^ inhibitors sotorasib and adagrasib resulted in response rates of about 40% in lung cancer patients (10, 11), various mechanisms of resistance have been observed, including secondary KRAS mutations (12–17). Furthermore, KRAS^G12C^ only accounts for approximately 12% of all KRAS mutations (8, 9, 18, 19). While direct KRAS^G12C^ inhibitors have proven that KRAS is druggable with clinically meaningful responses, there remains an urgent need for innovative molecules that can (a) target non-KRAS^G12C^ mutations, and (b) overcome the many resistance mechanisms frequently observed with KRAS targeting.

Several studies have shown that mutant KRAS cooperates with the proto-oncogene c-Myc (MYC) in promoting and maintaining aggressive tumorigenesis through several mechanisms, including stimulation of inflammation, activation of pro-survival pathways, and suppression of apoptosis (20–22). Importantly, MYC upregulation has been found to be a key mediator in promoting resistance to KRAS inhibition (16, 23–25). MYC is a transcription factor that has critical roles in homeostasis and regulates about 15% of the genome (26). Importantly, MYC is regarded as a critical oncoprotein and is dysregulated in approximately 50%–70% of cancers (26). Several studies have shown that downregulation and/or inactivation of MYC can substantially inhibit tumorigenesis, making it a very attractive therapeutic target (27–30). Like most KRAS-mutant proteins, MYC does not have any approved targeted therapies despite its intensive characterization, which is in part due to its unstructured domains, inaccessible localization in the nucleus, and ubiquitous expression in healthy tissues (4). Current therapeutic strategies for targeting MYC include targeting of MYC/Max heterodimers, use of a dominant-negative MYC mimic, and targeting of downstream genes. (31–35). However, others have shown that targeting MYC alone may not be sustainable as it may result in toxicity, or cancer cells may quickly evolve to reactivate it (36–38).

KRAS activation can stabilize MYC either by initiating the phosphorylation of MYC at serine 62 via ERK1/2 signaling or by preventing the phosphorylation of MYC at threonine 58 via inhibition of GSK3β, which usually targets MYC protein for degradation (39). In KRAS-mutant pancreatic cancer, MYC stabilization can also occur following ERK1/2 inhibition via the activation of the alternative MEK5/ERK5 pathway (40). Inhibition of MYC can sensitize cancer cells to cytotoxins and promote tumor regression and increased survival in mice (34, 41). These data strongly imply that the dual suppression of mutant KRAS and MYC may lead to a synergistic anticancer effect. Indeed, several independent studies using transgenic mouse models have shown that losing both oncogenes can lead to a greater reduction in tumor burden and enhanced survival in breast and lung cancer (42, 43).

RNA interference–based (RNAi-based) therapies present a unique alternative strategy for targeting “undruggable” proteins like MYC and KRAS and operate through the RNA-induced silencing complex (RISC) (44). Briefly, cytosolic RISC unwinds delivered dsRNA and loads the guide strand to recognize and cleave complementary mRNA sequences (44). Although RNAi is sequence specific and potent, until recently it has faced several clinical obstacles, including in vivo instability, lack of tissue-specific delivery, off-target silencing effects, and immunogenicity (45). However, recent innovations in the RNAi therapeutics field have led to the adoption of receptor-targeting ligands conjugated to fully chemically modified siRNAs (45). These advances have helped overcome many physiologic barriers, leading to several clinically approved RNAi-based drugs that silence mRNA targets in the liver (46–48). Although similar ligand-conjugated RNAi approaches have yet to succeed in the context of cancer treatment, there are several developing platforms that show good safety profiles and antitumor efficacy signals (49–51). Here, we describe what we believe to be novel compositions of inverted RNAi molecules that exhibit unexpectedly potent cosilencing of MYC and KRAS. These inverted RNAi molecules showed up to a 40-fold improvement in inhibition of cancer cell viability. Importantly, these chimeric designs may be broadly applicable for cosilencing any two target genes of interest, which has far-reaching implications for cancer and beyond.

## Results

### Identification of potent, chemically modified MYC and pan-KRAS siRNAs.

To identify *MYC*-targeting siRNA sequences, we analyzed human and mouse *MYC* sequences for conserved regions. Using several open-source design tools, we identified 8 sequences with high predicted efficacy against *MYC* (Supplemental Table 1; supplemental material available online with this article; https://doi.org/10.1172/JCI187204DS1), which target the highly conserved open reading frames of human and mouse *MYC* (Supplemental Figure 1A). We initially evaluated these sequences as unmodified siRNAs with 3′-deoxythymidine (dTdT) overhangs.

We transfected these siRNAs into MIA PaCa-2 pancreatic carcinoma cells (KRAS^G12C^/WT) to identify the most potent based on reduced *MYC* mRNA and protein expression. Compared with a nontargeting negative control (NC) siRNA, sequences 1, 2, 4, 6, and 8 greatly reduced *MYC* mRNA levels by 24 and 48 hours after transfection (Supplemental Figure 1B), and MYC protein levels by 24 hours (Supplemental Figure 1C).

To confer drug-like properties to the siRNAs (52), we included 2′-*O*-methyl and 2′-fluoro modifications on the sugar moieties, and phosphorothioates at the 5′ and 3′ ends of each strand in order to avoid endo- and exonuclease degradation, respectively. These chemical modifications have been shown to reduce immunogenicity and off-target effects and increase stability in vivo without marked reductions in efficacy (45, 53). For initial screening, we chose a higher 2′-fluoro (Hi2F) design. We tested the chemically modified versions of our top 5 candidates from the unmodified screen using MIA PaCa-2 and A427 (KRAS^G12D^/WT) lung carcinoma cells. Compared with NC siRNA, modified MYC-targeting sequences 2 and 3 (Mseq2 and Mseq3) reduced up to 80% of *MYC* mRNA in both cell lines at 24, 48, and 72 hours (Supplemental Figure 2A) and MYC protein levels at 72 hours (Supplemental Figure 2B).

To assess antitumor activity, we evaluated siRNA transfection on spheroid formation to simulate the tumor microenvironment. Compared with NC siRNA, Mseq2 and Mseq3 dramatically reduced spheroid density in both cell lines (Supplemental Figure 2C). These data demonstrate that our modified MYC siRNAs strongly silence *MYC* expression and significantly reduce tumorigenic potential.

Previous efforts in our laboratory led to the development of potent unmodified pan-KRAS siRNAs (54). Although these siRNAs demonstrated preclinical efficacy when delivered in nanoliposomes (54), the use of ligand-directed, fully chemically modified siRNAs has recently reshaped the RNAi field (45, 51, 52). By modifying these pan-KRAS siRNAs with a high proportion of 2′-*O*-methyl modifications (Hi2OMe), which confers improvements in metabolic stability within the endosomal compartment (55), we found that the KRAS siRNAs (Kseq2 and Kseq3) retained potent RNAi activity in several cancer cell lines (Supplemental Figure 2, D and E).

### Cotargeting with KRAS and MYC siRNAs reduces tumorigenic properties in vitro.

Mutant KRAS signaling stabilizes and hyperactivates MYC via ERK1/2, leading to MYC accumulation and sustained pro-tumorigenic signaling (Supplemental Figure 3A) (40). Simultaneous inactivation of these oncogenes has been shown to synergistically decrease tumor progression (42, 43).

We evaluated the effects of RNAi-mediated KRAS and MYC silencing on tumorigenesis. Compared with NC siRNA and individual KRAS or MYC siRNAs, equimolar combinations of KRAS and MYC siRNAs resulted in significantly reduced spheroid formation in MIA PaCa-2 cells (Supplemental Figure 3B). Similar results were observed in A427, H441 (lung carcinoma; KRAS^G12V^/WT), and HCT116 (colorectal carcinoma; KRAS^G13D^/WT) cells, and the combination of Kseq2 and Mseq2 siRNAs consistently performed as the most effective treatment (Supplemental Figure 3C). Our results demonstrate that dual siRNA-mediated silencing of KRAS and MYC is highly effective at preventing tumorigenesis beyond either siRNA alone across several cancer types and common KRAS mutations.

### Inverted multivalent chimeras potently cotarget MYC and KRAS oncogenes.

**Figure 1:**
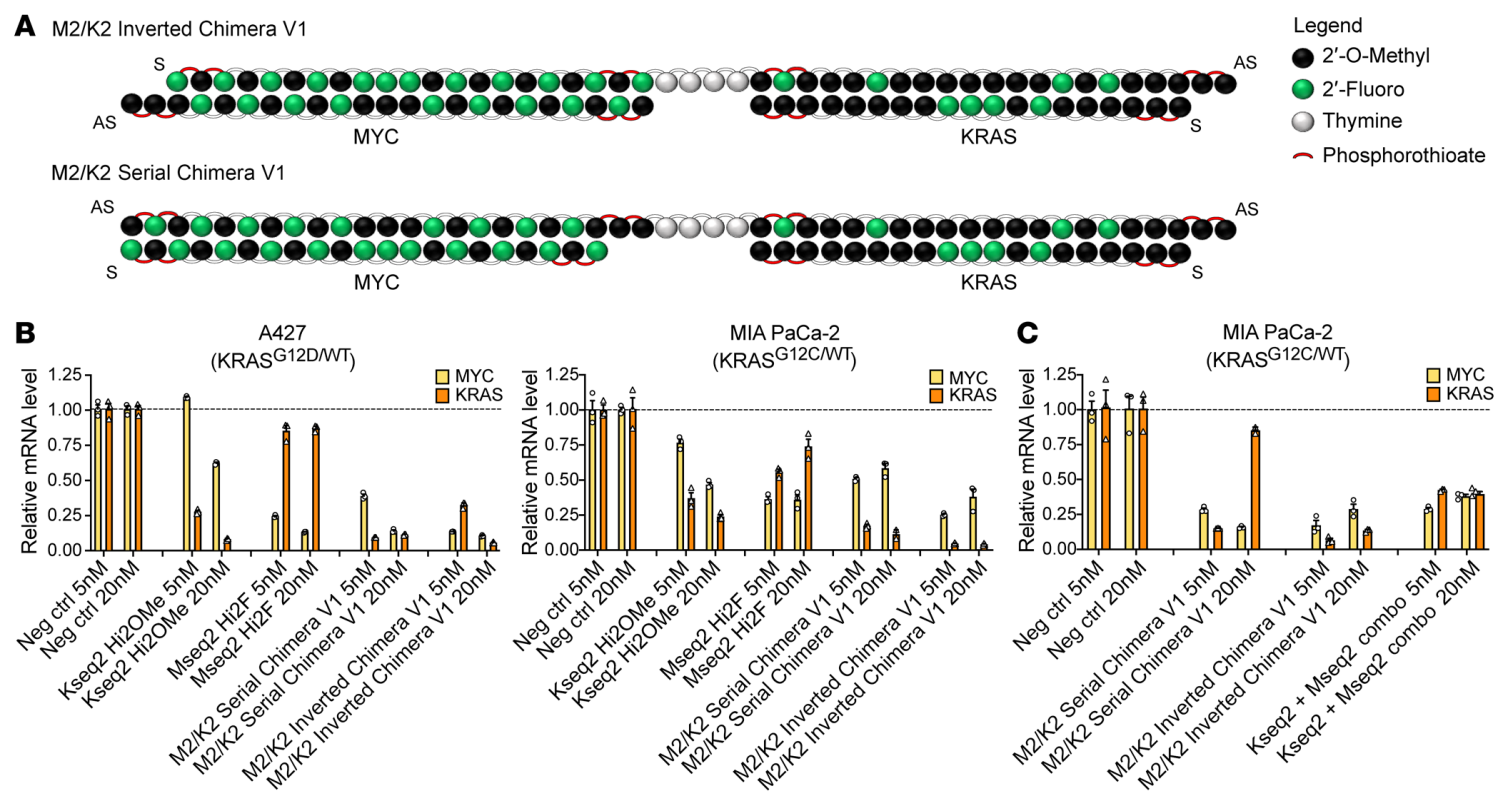
Design and in vitro activity of MYC/KRAS chimeric siRNAs. (**A**) Structures of the inverted and serial conformations of a MYC/KRAS cotargeting chimeric siRNA. In the inverted conformation (M2/K2 Inverted Chimera V1), the MYC siRNA passenger (sense, S) strand is linked via a d(T)_4_ bridge to the KRAS siRNA guide (antisense, AS) strand. In the serial conformation (M2/K2 Serial Chimera V1), the MYC siRNA guide (antisense) strand is linked via a d(T)_4_ bridge to the KRAS siRNA guide (antisense) strand. (**B** and **C**) Relative *MYC* and *KRAS* mRNA expression by RT-qPCR after siRNA treatment at 5 and 20 nM for 48–72 hours in A427 and MIA PaCa-2 cells. In conditions with MYC plus KRAS cotransfection, each of the MYC and KRAS siRNAs was transfected at the indicated dose. Data are shown as the mean ± SEM.

To ensure equimolar targeting of 2 siRNAs, we considered that phosphodiester bridges can confer “prodrug”-like activity in the plasma and allow for endonucleolytic metabolism within the target tissue (56). We developed 2 conformations of the KRAS and MYC chimera using a DNA bridge consisting of four 2′-deoxythymidines: a “serial” conformation linking the MYC and KRAS guide strands, and an “inverted” conformation linking the MYC passenger to the KRAS guide strand (Figure 1A). Mseq2 Hi2F and Kseq2 Hi2OMe modified siRNAs were used in preliminary chimera designs (M2/K2 Chimera Version 1 [V1]).

To test the chimeric siRNA designs, we evaluated equimolar transfections of the siRNAs at various doses and time points. Although both chimeric designs improved potency, we found that M2/K2 Inverted Chimera V1 was more potent at silencing both *MYC* and *KRAS* than M2/K2 Serial Chimera V1 (Figure 1B). M2/K2 Inverted Chimera V1 was also consistently as effective as or better than codelivery of individual siRNAs (Figure 1C and Supplemental Figure 3D).

The enhanced potency of the inverted chimeric design was validated in additional cell lines (Supplemental Figure 4, A and B). Notably, the inverted chimeric design was far more potent at silencing *MYC* and *KRAS* beyond that seen with the individual siRNAs. For example, at 5 nM, either of the MYC or KRAS siRNAs resulted in about 70% silencing; however, the inverted chimeric design led to more than 90% target silencing of *KRAS* (Figure 1B and Supplemental Figure 4, A and B). Similar observations were made at the protein level, where the inverted chimeric design showed improved silencing compared with the serial chimeric design (Supplemental Figure 4C). Together, these data demonstrate that the inverted chimeric siRNA is more potent than its individual siRNA components in combination, and that the orientation of the individual components affects the chimera’s efficacy.

### Chimeric siRNAs are metabolized primarily in endosomes.

**Figure 2:**
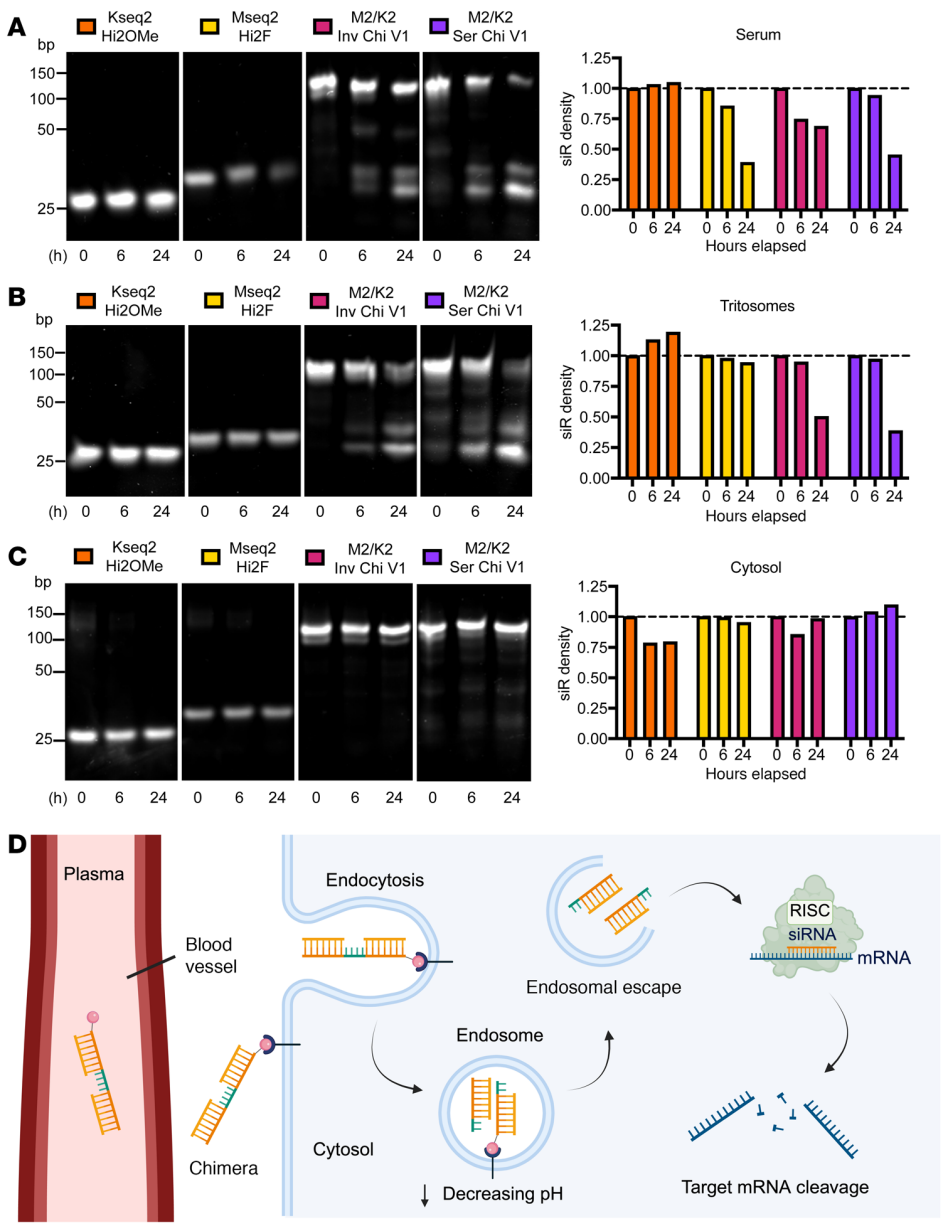
Stability of MYC/KRAS chimeric siRNAs in different cellular conditions. (**A**) Evaluation of siRNA stability in serum. Ten micromolar of the MYC Hi2F, KRAS Hi2OMe, M2/K2 Inverted Chimera V1 (M2/K2 Inv Chi V1), and M2/K2 Serial Chimera V1 (M2/K2 Ser Chi V1) siRNAs were incubated in 50% FBS for 0, 6, and 24 hours. (**B**) Evaluation of siRNA stability in tritosomes. Four micromolar of the MYC Hi2F, KRAS Hi2OMe, M2/K2 Inv Chi V1, and M2/K2 Ser Chi V1 siRNAs were incubated in acidified rat liver tritosomes for 0, 6, and 24 hours. (**C**) Evaluation of siRNA stability in cytosol. Ten micromolar of the MYC Hi2F, KRAS Hi2OMe, M2/K2 Inv Chi V1, and M2/K2 Ser Chi V1 siRNAs were incubated in rat liver cytosol for 0, 6, and 24 hours. (**A**–**C**) Quantification of relative band intensities is included to the right, which were normalized to the 0 hours time point for each siRNA. Images are representative of experiments conducted 2 times. (**D**) Schematic of siRNA metabolism following in vivo administration. Created with BioRender.

The plasma half-life of ligand-conjugated siRNAs ranges from around 15 to 90 minutes, depending on variables such as the conjugated ligand, linker, oligonucleotide modifications, and delivery routes (50, 57). Upon systemic administration, ligand-conjugated siRNAs travel through the bloodstream and are directed to their intended target receptor. Upon receptor engagement, the ligand and siRNA payload are internalized via clathrin-mediated endocytosis, and the latter eventually escapes into the cytosol to become incorporated into the RISC complex and elicit RNAi-mediated target mRNA degradation (53). In the endosomal compartment, siRNAs are exposed to nucleolytic enzymes, which can lead to degradation (58). To assess where these chimeric siRNAs are metabolically processed, we incubated the individual MYC and KRAS siRNAs and the two M2/K2 chimera designs in plasma, endosomal, and cytosolic conditions for up to 24 hours. First, we tested stability in 50% serum and found that both chimera designs had minimal metabolic processing after 6 hours, suggesting they would largely remain intact upon target tissue exposure in vivo (57). By 24 hours, the serial chimera had undergone increased cleavage compared with the inverted chimera (Figure 2A). Additionally, comparing the two chemical modification patterns, the MYC Hi2F siRNA degraded more quickly than the KRAS Hi2OMe siRNA, supporting the idea of increased siRNA stability with increased 2′OMe content (59). Next, we incubated the siRNAs in acidified rat liver tritosomes as a proxy for endosomes, which undergo a decrease in pH as they become lysosomes (58). While the individual MYC and KRAS siRNAs remained relatively unprocessed, both chimeras underwent cleavage at the thymidine bridge by 24 hours (Figure 2B). The entire chimeric structure was disrupted by 48 hours (Supplemental Figure 5A). To assess whether cleavage was due to the acidic pH within the endosome, we incubated the siRNAs in an acidic buffer without tritosomes and found that the chimeras remained stable for up to 48 hours (Supplemental Figure 5B), suggesting that the metabolism of the chimeras is through endonucleolytic cleavage. We incubated the siRNAs with rat liver cytosol and found they remained mostly unprocessed (Figure 2C).

During the RNAi process, the RNase type III enzyme Dicer can process long dsRNA into 21- to 23-base-pair fragments (44). The chimeric siRNA constructs investigated here are 46 nucleotides long, i.e., twice as long as the dsRNAs produced by Dicer. Therefore, we tested whether the chimera could also serve as a substrate for Dicer. We directly treated the siRNAs with recombinant human Dicer and found that the chimeric molecules were not processed (Supplemental Figure 6A). We also evaluated knockdown efficiency by comparing dose-response and kinetics between a parental HEK293T cell line and a Dicer CRISPR knockout line (HEK293T NoDice). We found no significant decrease in knockdown efficacy for either chimeric design (Supplemental Figure 6, B and C). Based on these observations, we conclude that both chimeric siRNA designs remain intact in plasma conditions and are predominantly metabolized within the endosomal compartment upon receptor-mediated endocytosis (Figure 2D).

### Chimeric bridge cleavage results in more potent 5′-guide overhangs.

Argonaute2 (Ago2) is the key enzyme in RISC responsible for mediating RNAi (45). Ago2 interacts with guide RNA through the MID domain (binds the 5′ end of a guide RNA), PIWI domain (induces cleavage), and PAZ domain (anchors the 3′ end of a guide RNA) (45). Initial modeling assessed the possibility of the full inverted chimeric strand (i.e., the passenger strand of Mseq2 and the guide strand of Kseq2 linked by the thymidine bridge) getting loaded into Ago2 opposite the *KRAS* target strand. However, using structural modeling, we determined that weaving the linker portion out of the MID/PIWI binding cleft while avoiding clashes with Ago2 side chains and/or target strand residues is nearly impossible (Supplemental Figure 7, A and B). This suggests that it is unlikely that the full chimeric construct is accommodated inside Ago2 opposite the target mRNA, with its phosphodiester moiety between dT and the 5′-most U of Kseq2 bound in the MID binding pocket like a 5′-terminal phosphate. Instead, we determined it is more likely that the KRAS guide strand gets loaded into Ago2 after its 5′-end undergoes metabolic processing.

To determine the identity of the metabolic products following endosomal processing, we incubated the M2/K2 Inverted Chimera in endosomal conditions for 48 hours and analyzed the samples with liquid chromatography–mass spectrometry (LC-MS). We detected nearly every potential metabolic product with dT overhangs, confirming cleavage at the thymidine bridge (Supplemental Figure 8, A and B, and Supplemental Table 2). Owing to the length of incubation and differences in 5′ and 3′ exonuclease degradation dynamics, it is likely that the proportions of metabolic products in our sample are not equivalent to exact cellular conditions, as these molecules may undergo further processing to remove the overhangs. Based on these data, we evaluated whether the metabolic products of the cleaved thymidine bridge (5′-dT overhangs) could explain the potency of the chimeric designs.

**Figure 3:**
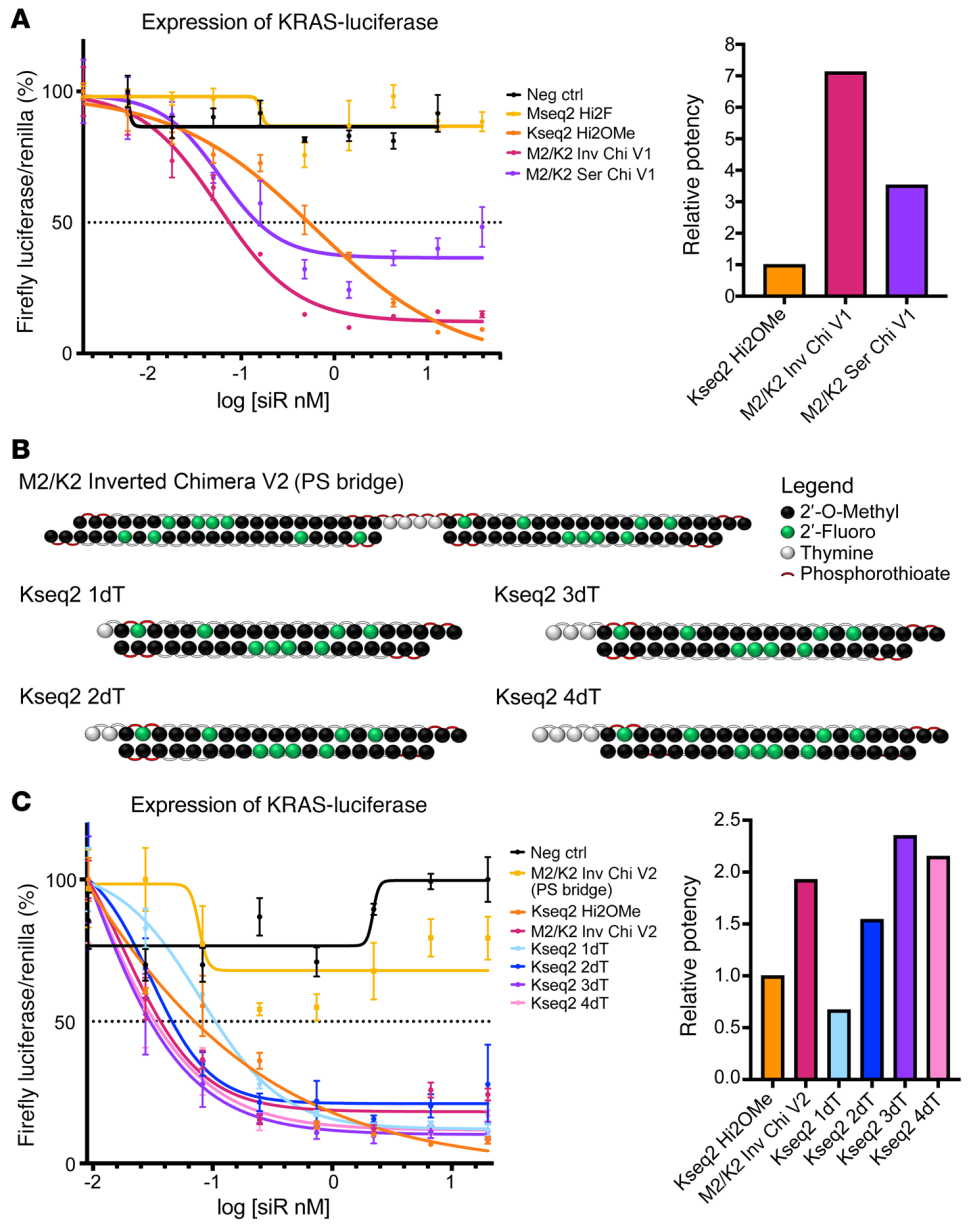
Characterization of MYC/KRAS chimeric siRNA mechanism of action. (**A**) Dose-response curves (left) and relative ED_50_ values (right, calculated as ED_50_ of siRNA divided by ED_50_ of Kseq2 Hi2OMe) of KRAS–firefly luciferase expression in A-431 KRAS-knockout cells treated with the nontargeting negative control (NC) siRNA, MYC Hi2F, KRAS Hi2OMe, M2/K2 Inverted Chimera V1 (M2/K2 Inv Chi V1), and M2/K2 Serial Chimera V1 (M2/K2 Ser Chi V1). All firefly luciferase luminescence values were normalized with Renilla luciferase luminescence and expressed as a percentage. Data are representative of 3 replicates, and error bars represent SEM. (**B**) Structures of M2/K2 Inverted Chimera V2 with a fully phosphorothioate-modified bridge that renders it uncleavable, and the 4 possible iterations of the metabolized Kseq2 siRNA with 1, 2, 3, or 4 dT overhangs. (**C**) Dose-response curves (left) and relative ED_50_ values (right, calculated as ED_50_ of siRNA divided by ED_50_ of Kseq2 Hi2OMe) of KRAS–firefly luciferase expression in A-431 KRAS-knockout cells treated with the NC siRNA, M2/K2 Inverted Chimera V2 with a fully phosphorothioate-modified thymidine bridge [M2/K2 Inv Chi V2 (PS bridge)], KRAS Hi2OMe, M2/K2 Inverted Chimera V2 (M2/K2 Inv Chi V2), Kseq2 1dT, Kseq2 2dT, Kseq2 3dT, and Kseq2 4dT. All firefly luciferase luminescence values were normalized with Renilla luciferase luminescence and expressed as a percentage. Data are representative of 2 replicates, and error bars represent SEM.

We used an A-431 KRAS CRISPR knockout line stably transduced with a KRAS–firefly luciferase reporter system to evaluate knockdown efficiency on a 10-point dose-response curve (60). We observed that both chimeric designs decreased KRAS expression more potently than the single KRAS siRNA, and that the inverted chimera showed the highest potency (Figure 3A). To evaluate all possibilities of the thymidine bridge cleavage, with two 2′-deoxythymidine (2dT) 5′-terminal overhangs being the most likely (56), we tested iterations of Kseq2 with 5′-dT overhangs at each thymidine position on the guide strand. We also included a “non-cleavable” thymidine bridge with phosphorothioate modifications throughout to confirm that the chimera must be cleaved for RNAi activity (Figure 3B). As expected, the fully modified “non-cleavable” thymidine bridge showed essentially no knockdown (Figure 3C). Unexpectedly, we found that Kseq2 with 5′-dT overhangs on the guide strand performed better than Kseq2 alone, with increasing potency directly correlated with the addition of each dT, although the increased potency plateaued at 3 dT.

**Figure 4:**
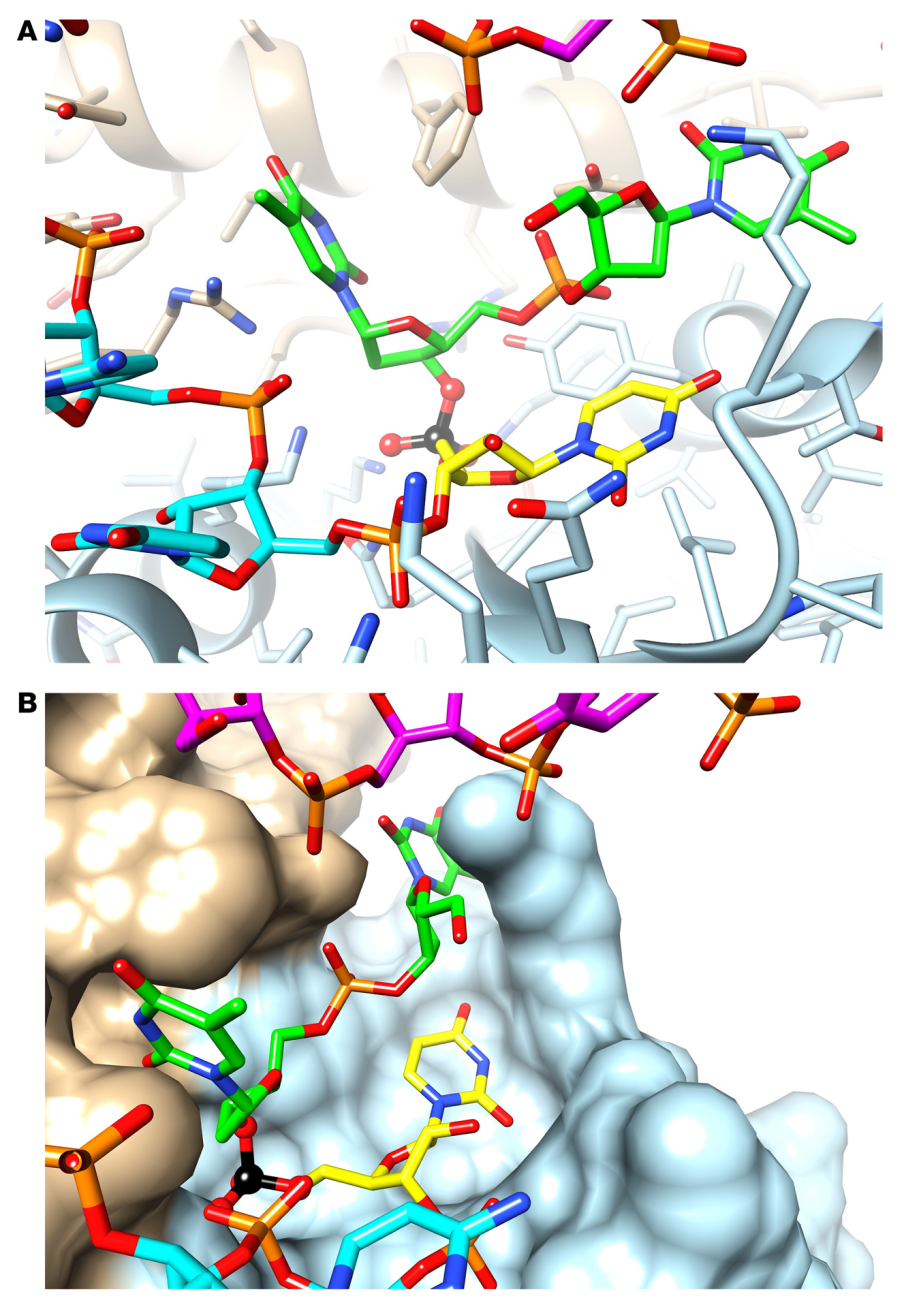
Model of inverted chimeric siRNA cleavage product within Ago2 complex. (**A**) Representation of the active KRAS guide strand with a 2dT 5′-overhang. KRAS guide siRNA carbon atoms are in cyan, dTdT carbon atoms are in light green, carbon atoms of the 5′-terminal U1 of the KRAS guide strand are in yellow, and carbon atoms of amino acids from the Ago2 MID and PIWI domains are in light blue and tan, respectively. (**B**) Model depicting the KRAS guide strand bound to Ago2 with the protein shown in a surface representation. Ago2 MID and PIWI domain residues are colored in light blue and tan, respectively, the phosphorus atom of the “former” 5′-terminal phosphate lodged at the MID Lys/Arg/Gln/Tyr binding pocket is highlighted in black, and the strand with carbon atoms colored in purple is the targeted *KRAS* mRNA.

It is possible that the potency is due to a shift in the seed region of the guide strand. To test whether this was the reason for the increase in potency, we created two Mseq2 and two Kseq2 siRNAs with overhangs that were perfectly complementary to the target mRNA (Supplemental Figure 9A). We then conducted 10-point dose-response assays to compare the effect on cell viability. We found that Mseq2 2dT was still more potent than Mseq2 dTG, and there was total abolishment of activity with Mseq2 dTGA (Supplemental Figure 9B). We found a similar pattern when testing Kseq2 dGT and dGTG using our KRAS-luciferase reporter (Supplemental Figure 9C). Based on these data, we do not believe that the improvement in potency from 5′-thymidine overhangs is related to a shift in the seed region, and instead, the guide strand of Kseq2 with a 2dT overhang at the 5′-end is becoming incorporated into Ago2 to induce mRNA silencing, whereby the 2dT overhang fits into the MID domain binding pocket (Figure 4, A and B). Thus, the phosphodiester linkage (charge –1) between Kseq2 and 2dT sits in the MID domain binding pocket normally occupied by the 5′-terminal phosphate (charge –2) of guide siRNA. The 2 thymidines protrude into the cleft between the MID and PIWI domains and can be accommodated between the guide and target strands.

### Inverted MYC/KRAS chimeras synergistically target KRAS mutant cancers.

**Figure 5:**
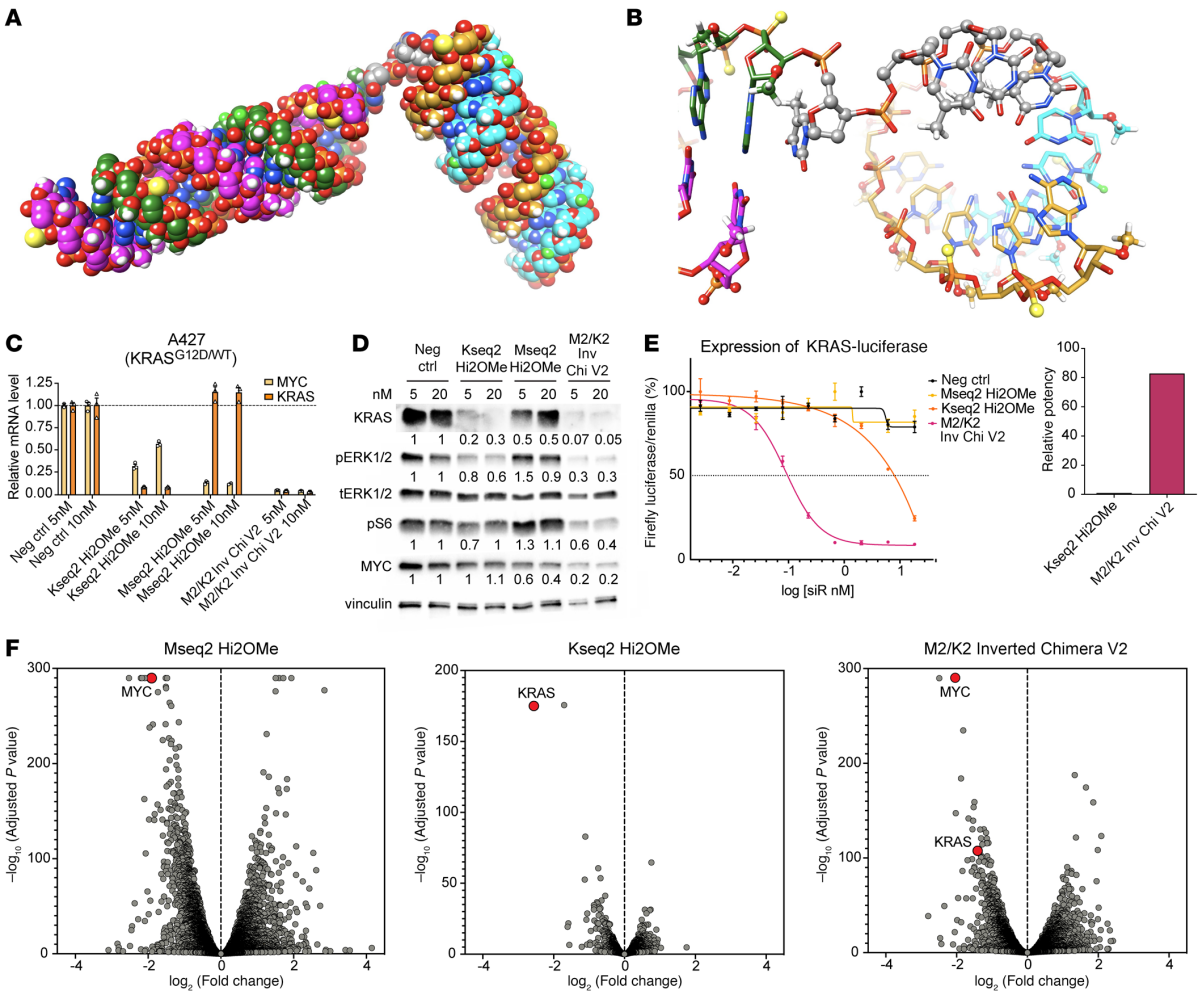
Characterization of MYC/KRAS inverted chimeric siRNA with enhanced 2′OMe chemical modification. (**A**) 3D space-filling model of the fully modified M2/K2 Inverted Chimera V2. Carbon atoms of the MYC guide strand are in magenta, and carbon atoms of the passenger strand are in green. Carbon atoms of the KRAS guide strand are in cyan, and carbon atoms of the passenger strand are in gold. The thymidine bridge is shown with carbon atoms in gray, 2′-fluorine atoms are light green, and phosphorothioate sulfur atoms are yellow. (**B**) Ball-and-stick model showing a portion of the inverted chimeric siRNA, with the KRAS G:P duplex viewed along the helical axis and carbon atoms of the kinked d(T)_4_ bridge highlighted as gray spheres. The color code is the same as in **A**. (**C**) Relative *MYC* and *KRAS* expression by RT-qPCR in A427 cells following treatment with the negative control. siRNA, MYC Hi2OMe, KRAS Hi2OMe, and M2/K2 Inverted Chimera V2 at 5 and 10 nM for 72 hours. Error bars represent SEM. (**D**) KRAS, phospho-ERK1/2, total ERK1/2, phospho-S6, and MYC expression by Western blot in A427 cells following treatment with the negative control siRNA, MYC Hi2OMe, KRAS Hi2OMe, and M2/K2 Inverted Chimera V2 at 5 and 20 nM for 72 hours. Relative expression values are shown below each band for KRAS, phospho-ERK1/2, phospho-S6, and MYC. (**E**) Representative dose-response curves and ED_50_ values of KRAS–firefly luciferase expression in A-431 KRAS-knockout cells treated for 4 days with the NC siRNA, MYC Hi2OMe, KRAS Hi2OMe, and M2/K2 Inverted Chimera V2. All firefly luciferase luminescence values were normalized with Renilla luciferase luminescence and expressed as a percentage. Error bars represent SEM. (**F**) RNA sequencing volcano plots showing all genes upregulated and downregulated in comparison with negative control conditions following treatment of A427 cells with indicated siRNAs at 20 nM for 24 hours.

To further stabilize the inverted chimera (59), we evaluated the Hi2OMe chemical modification pattern on the MYC siRNAs (Supplemental Figure 10A). Knockdown efficiency of MYC siRNAs with Hi2F and Hi2OMe modification patterns was similar across several cell lines, with Mseq2 Hi2OMe being the most potent (Supplemental Figure 10B). We incorporated this design into the M2/K2 inverted chimeric siRNA to generate M2/K2 Inverted Chimera Version 2 (V2) (Supplemental Figure 10C). Comparing M2/K2 V1 and V2 inverted chimera designs at low doses showed nearly equipotent levels of MYC and KRAS silencing (Supplemental Figure 10D), suggesting that M2/K2 Inverted Chimera V2 experiences no loss of potency while having improved metabolic stability conferred by additional 2′OMe modifications in vivo. Modeling full M2/K2 Inverted Chimera V2 in silico revealed that the thymidine bridge is flexible and that the linked siRNAs likely have a dynamic orientation to each other (Figure 5, A and B).

We found that M2/K2 Inverted Chimera V2 has far more potency than the individual siRNAs across several doses and cell lines, particularly for KRAS (Figure 5C and Supplemental Figure 11A). Similarly, we observed a clear dose-response in MIA PaCa-2 and A427 cell lines on a protein level, which showed that M2/K2 Inverted Chimera V2 substantially reduced both MYC and KRAS protein levels and MAPK signaling (evaluated by ERK1/2 phosphorylation) compared with individual siRNAs (Figure 5D and Supplemental Figure 11B). Using our KRAS-luciferase reporter, we also observed that M2/K2 Inverted Chimera V2 was 80-fold more potent than the Kseq2 siRNA alone (Figure 5E). To examine the off-target effects of our siRNAs, we conducted RNA sequencing on the A427 cells after treating with the negative control siRNA, Kseq2 Hi2OMe siRNA, Mseq2 Hi2OMe siRNA, and M2/K2 Inverted Chimera V2 after 24 hours. We found that MYC and KRAS were among the top downregulated genes in their respective targeted siRNA treatments, and both were strongly downregulated by the chimera (Figure 5F). Based on these data, we concluded that the siRNAs are specifically targeting the genes of interest.

**Figure 6:**
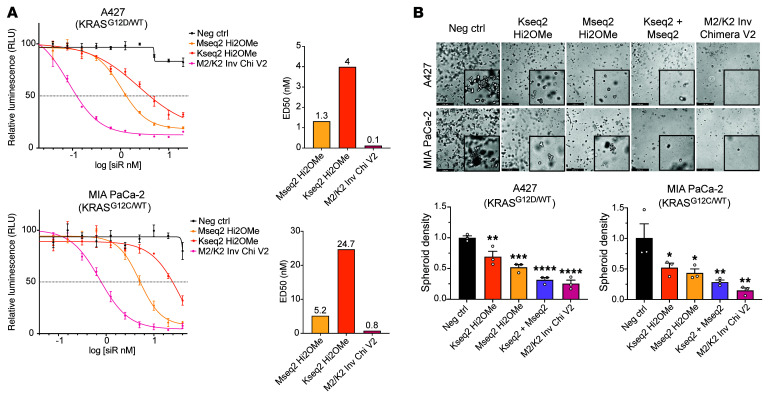
Effects of M2/K2 inverted chimeric siRNA on cancer cell viability. (**A**) Representative dose-response curves and ED_50_ values for MIA PaCa-2 and A427 cells treated for 6 days with the negative control siRNA, MYC Hi2OMe, KRAS Hi2OMe, and M2/K2 Inverted Chimera V2. ED_50_ values are shown in nanomolar above the respective bar in the bar graphs on the right. Data are representative of 3 replicates, and error bars represent SEM. (**B**) Representative images and quantification of spheroids in a tumorigenesis assay in Matrigel with A427 and MIA PaCa-2 cells. Images were taken with a ×5 microscope objective. Scale bars: 498 μm. Error bars represent SEM. One-way ANOVA was used for statistical comparisons. *P < 0.05, **P < 0.01, ***P < 0.001, ****P < 0.0001.

To evaluate the phenotypic effects of siRNA-mediated dual knockdown of MYC and KRAS, we conducted a dose-response assay and found that the inverted chimeric design substantially lowered the ED_50_ in these KRAS-dependent cell lines more than 20- to 40-fold, going from low-nanomolar doses for individual MYC or KRAS siRNAs down to as low as 100 pM for the Inverted Chimera V2 design (Figure 6A). The chimeric siRNA’s improved potency was also observed in small-cell lung cancer lines that are MYC-dependent with wild-type KRAS. As expected, KRAS siRNAs had almost no inhibitory effect (Supplemental Figure 12). These results are likely due to the combined effect of downregulating MYC through direct target RNA engagement and through its upstream regulator, KRAS. Next, we evaluated the effects of cotargeting MYC and KRAS on spheroid formation. In comparison with individual Mseq2 or Kseq2 siRNAs, the cells treated with the combination of the individual siRNAs, or M2/K2 Inverted Chimera V2, showed significantly diminished spheroid formation (Figure 6B). Taken together, these results show that the optimized V2 inverted chimeric siRNA demonstrates marked improvements in targeting both MYC and KRAS, resulting in attenuated MAPK signaling and synergistic inhibition of cancer cell viability.

### EGFR-targeting ligand enables specific uptake into tumors.

**Figure 7:**
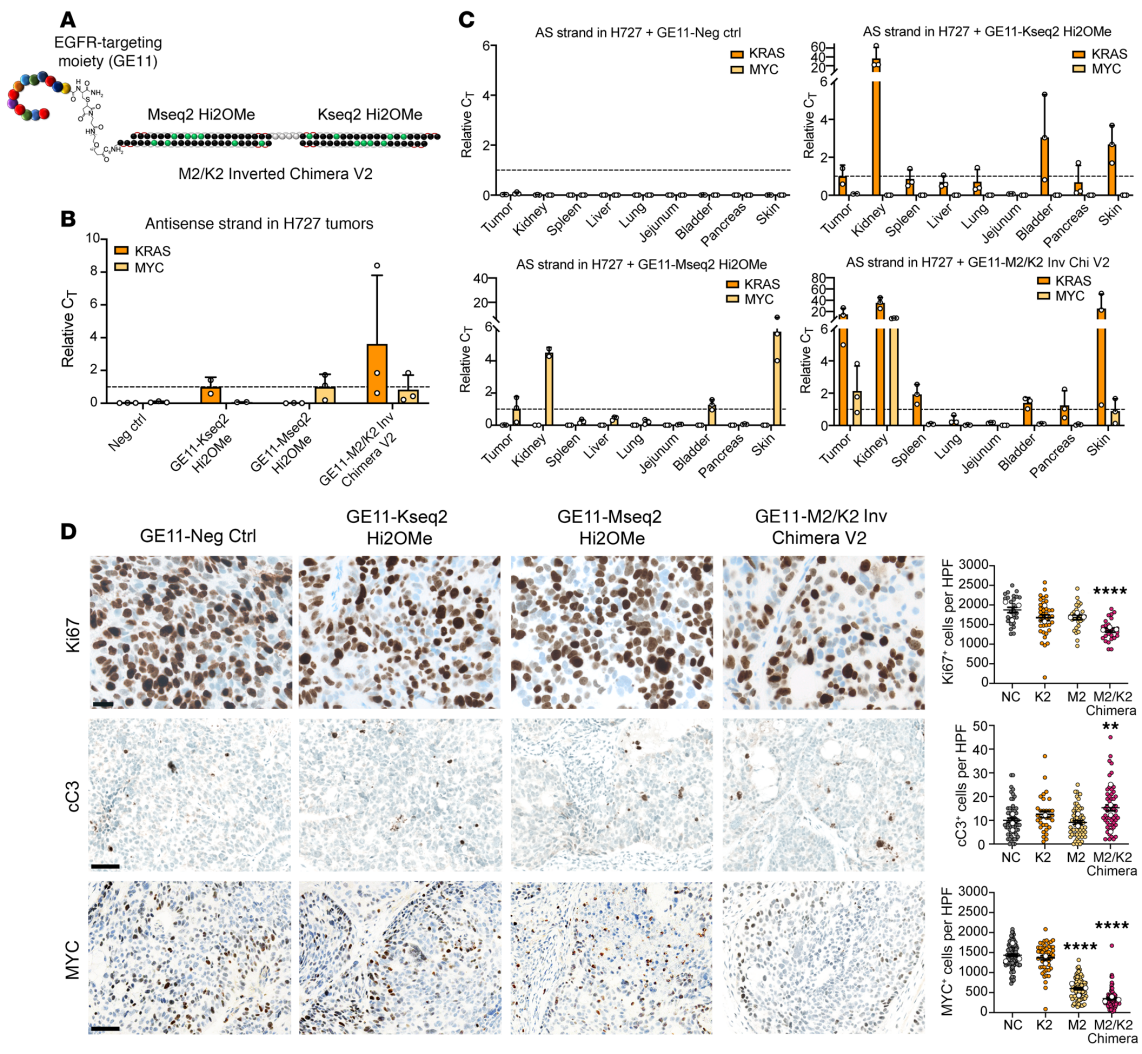
Characterization of receptor-targeting ligand GE11. (**A**) Structure of GE11-conjugated M2/K2 Inverted Chimera V2 (at the 3′ end of the guide strand). (**B**) Relative abundance values of the MYC and KRAS antisense (AS; guide) strands in aggregate tumors of each treatment group. Relative values for the MYC guide strand were normalized to the GE11-MYC siRNA treatment group, and relative values for the KRAS guide strand were normalized to the GE11-KRAS siRNA treatment group. Error bars represent SEM. (**C**) Relative AS abundance values of the MYC and KRAS guide strands in aggregate tumors, kidneys, spleen, lung, jejunum, bladder, pancreas, and skin of each treatment group. Relative values for the MYC guide strand were normalized to the GE11-MYC siRNA treatment group, and relative values for the KRAS guide strand were normalized to the GE11-KRAS siRNA treatment group. Error bars represent SEM. (**D**) Left: Representative images of Ki67, cleaved caspase-3 (cC3), and MYC staining in paraffin-embedded sections of H727 tumors treated for 7 days with siRNAs. Ki67 scale bar: 20 μm; cC3 and MYC scale bars: 50 μm. Right: Quantification of the positive cells per high-power field (HPF) in sections of H727. Error bars represent SEM. Unpaired 1-tailed *t* test corrected for multiple comparisons with the Bonferroni method was used for statistical comparisons. **P < 0.01, ****P < 0.0001.

Given the success of GalNAc-conjugated chemically optimized siRNAs (53, 59), which represent the overwhelming majority of recently approved clinical siRNA therapeutics, we evaluated whether a ligand-conjugated approach could target tumor cells and obviate the need for a nanoparticle-based carrier. Because the epidermal growth factor receptor (EGFR) is highly expressed in nearly all carcinomas and capable of receptor recycling after endocytosis (61), we sought to determine whether an EGFR-targeting ligand could enable tumor-directed chimera delivery. The EGFR ligand, GE11, is a 12–amino acid peptide discovered using phage display for EGFR that does not induce mitogenic signaling (62), and several independent groups have shown that nano-formulations of GE11 can target EGFR-expressing tumors (63, 64). Our laboratory has published work formally evaluating whether direct linker-mediated conjugation of GE11 to oligonucleotides could facilitate targeted RNAi delivery (65). In previous experiments, compared with unconjugated siRNAs, GE11-conjugated siRNAs showed an approximately 15-fold increase in uptake by EGFR-expressing cancer cells, likely due to receptor-mediated endocytosis. FACS sorting on samples from a xenograft model injected subcutaneously with conjugated siRNAs demonstrated robust tumor targeting, with approximately 90% of cancer cells taking up the siRNA. To test the specificity of the GE11 ligand, we used an amine-based conjugation strategy to covalently link GE11 with a C-terminal cysteine to a triethylene glycol linker and the 3′ end of the guide strand (66) of the MYC Hi2OMe siRNA (Figure 7A). Athymic nude mice bearing subcutaneous H727 (KRAS^G12V^/WT; lung carcinoid) tumors were randomly assigned to 4 treatment groups (*n* = 3 mice per group): 1: GE11–negative control (which is a non-targeting inert siRNA); 2: GE11–Mseq2 Hi2OMe; 3: GE11–Kseq2 Hi2Ome; and 4: GE11–M2/K2 Inverted Chimera V2. Once tumors reached about 75 mm^3^, GE11-conjugated siRNAs (groups 1–3: 5 mg/kg siRNA; group 4: 10 mg/kg chimera to yield 5 mg/kg of each siRNA) were injected subcutaneously twice weekly. Subcutaneous injection of ligand-conjugated siRNAs has been previously shown to perform better in vivo than intravenous administration (53) and is the preferred method of administration clinically. Following administration, the siRNAs will diffuse slowly from the injection site into the plasma, which will reach their target tissue via circulation (53). After 1 week of treatment (or 2 doses), tumors and several somatic tissues were collected. Following RNA isolation, using stem-loop quantitative reverse transcription PCR (RT-qPCR) to detect individual guide strands, we confirmed that the GE11-mediated delivery platform delivered MYC and KRAS siRNAs to the tumor. Interestingly, we observed a dramatic increase in the abundance of the KRAS guide strand in the MYC/KRAS chimeric siRNA–treated group, suggesting that the chimeric design has improved metabolic stability (Figure 7B). Similar to our previously published work, we observed the presence of the guide strands in other highly EGFR-expressing tissues, such as the skin and bladder (although we did not observe any adverse effects in the treated mice). Due to the hydrophilic nature of the modified siRNAs, clearance through the kidney was expected and observed (Figure 7C).

To evaluate for biological effects on a protein level, we performed tumor immunohistochemistry (IHC). While both KRAS and MYC siRNA–treated groups showed a decrease in Ki67, M2/K2 Inverted Chimera V2 treatment resulted in a more significant reduction, consistent with the inhibitory effects on proliferation via on-target downregulation of MYC and KRAS. Only the inverted chimeric siRNA resulted in a small but significant increase in cleaved caspase-3. Consistent with potent on-target regulation, treatment with MYC siRNAs resulted in a 54% reduction in MYC IHC staining, and MYC/KRAS inverted chimeric siRNAs resulted in a highly significant 76% reduction (Figure 7D). Taken together, these results demonstrate an effective, systemic EGFR-directed ligand-conjugated platform for cancer delivery. Additionally, the increased metabolic stability of the inverted chimeric design may further contribute to the improved effects on inhibition of proliferation and MYC expression.

### Ligand-conjugated inverted MYC/KRAS chimeras have potent antitumor activity.

**Figure 8:**
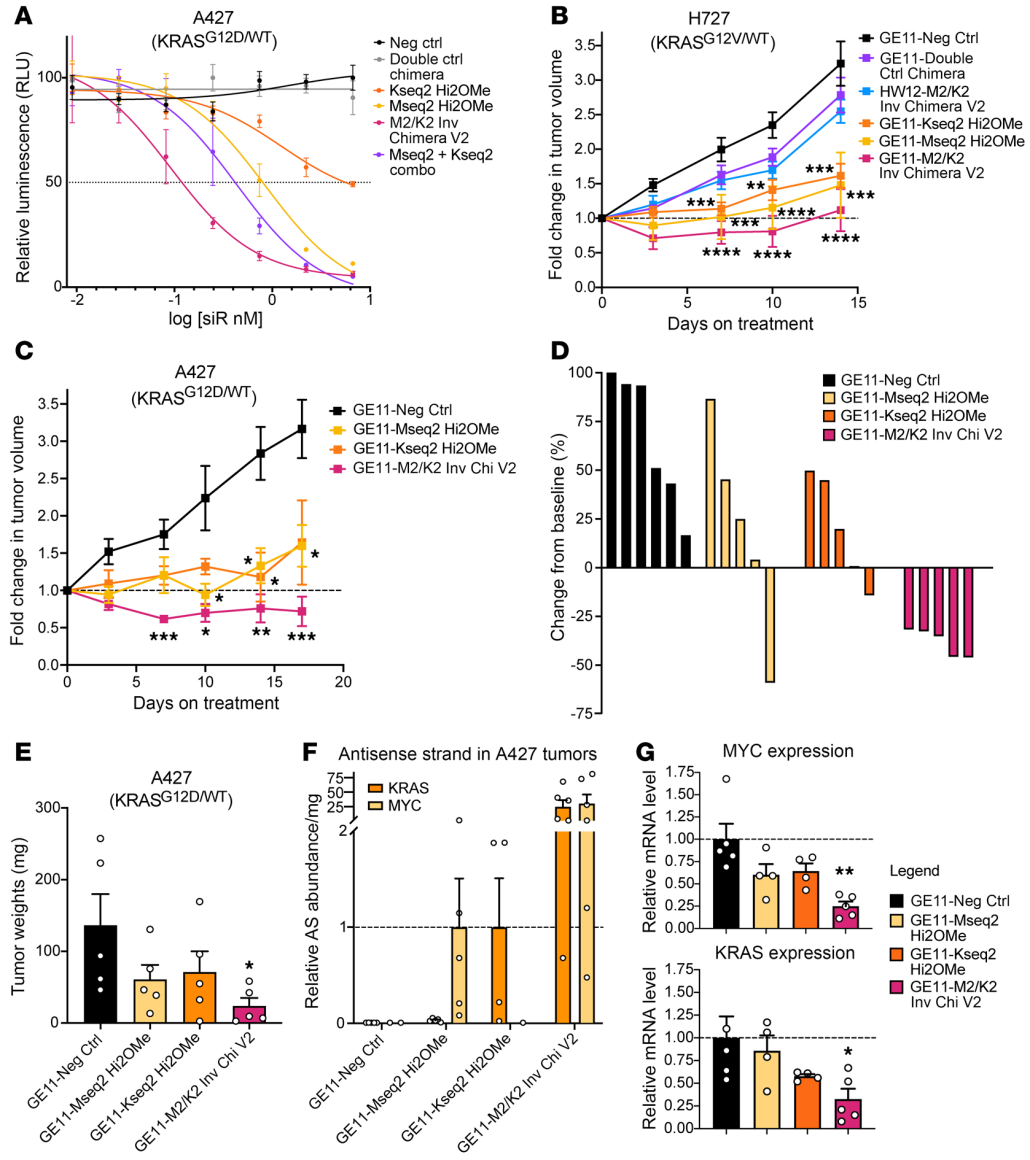
In vivo activity and efficacy of M2/K2 inverted chimeric siRNA. (**A**) Representative dose-response curves for A427 cells treated for 6 days with negative control siRNA, double-control siRNA, Mseq2 Hi2OMe, Kseq2 Hi2OMe, and M2/K2 Inverted Chimera V2. Error bars represent SEM. (**B**) Tumor growth curves showing average fold change in H727 tumor volume over 15 days (*n* = 10 for all treatment groups). Error bars represent SEM. Unpaired 1-tailed *t* test corrected for multiple comparisons using the Bonferroni method was used for statistical comparisons. (**C**) Tumor growth curves showing average fold change in A427 tumor volume over 21 days (*n* = 6 for GE11–Neg Ctrl, *n* = 5 for GE11–Mseq2 Hi2OMe, GE11–Kseq2 Hi2OMe, and GE11–M2/K2 Inverted Chimera V2). Error bars represent SEM. Unpaired 1-tailed *t* test corrected for multiple comparisons using the Bonferroni method was used for statistical comparisons. (**D**) Percentage change in A427 tumor volume for each mouse from baseline after 8 days of siRNA treatment. (**E**) Tumor mass in all treatment groups following cross-sectional necropsy at day 21 (*n* = 5 for all groups). Error bars represent SEM. Unpaired 1-tailed *t* test was used for statistical comparisons. (**F**) Relative abundance values of MYC and KRAS antisense (guide) strands per milligram of tumor of each treatment group. Relative values for MYC guide strand were normalized to the GE11-MYC siRNA treatment group, and relative values for the KRAS guide strand were normalized to the GE11-KRAS siRNA treatment group. Error bars represent SEM. (**G**) Relative *MYC* and *KRAS* mRNA expression in tumors of each treatment group (*n* = 5 for GE11–Neg Ctrl and GE11–M2/K2 Inverted Chimera V2 groups, *n* = 4 for GE11-Mseq2 and GE11-Kseq2 groups). Error bars represent SEM. Unpaired 1-tailed *t* test corrected for multiple comparisons using the Bonferroni method was used for statistical comparisons. *P < 0.05, **P < 0.01, ***P < 0.001, ****P < 0.0001.

To validate that the effect on cell viability was the result of specific gene targeting, we conducted dose-response assays in several cell lines comparing all treatment groups with an additional double-control chimera, which links 2 non-targeting siRNAs in the same configuration as M2/K2 Inverted Chimera V2. We found that the double-control chimeric siRNA had no effect on cancer cell viability (Figure 8A and Supplemental Figure 13A), further confirming that the potent decrease of cell viability following treatment of the MYC/KRAS inverted chimeric siRNA was due to specific knockdown of the genes of interest. To test the therapeutic effects of the conjugated siRNAs on tumor burden over time, athymic nude mice bearing subcutaneous H727 tumors were randomly assigned to the following treatment groups (*n* = 10 mice per group): 1: GE11–negative control; 2: GE11–Double-Control Chimera; 3: HW12–M2/K2 Inverted Chimera V2 (which contained a non-targeting version of GE11 [HW12] previously characterized by Gu and colleagues; ref. 62); 4: GE11–Mseq2 Hi2OMe; 5: GE11–Kseq2 Hi2OMe; and 6: GE11–M2/K2 Inverted Chimera V2. Once tumors reached about 75 mm^3^, mice were treated subcutaneously twice weekly (GE11-conjugated siRNA groups: 5 mg/kg siRNA; GE11-conjugated chimeric siRNA groups: 10 mg/kg chimera to yield 5 mg/kg of each siRNA). Compared with GE11–negative control siRNA treatment, we observed no significant tumor growth inhibition following treatment with GE11–Double-Control Chimera or HW12–M2/K2 Inverted Chimera V2, consistent with the chimeric structure having no efficacy on its own, as well as the requirement for the GE11 ligand to achieve effective tumor delivery. However, by day 7, we observed reduced tumor volumes following treatment with GE11–Mseq2 Hi2OMe (50%), GE11–Kseq2 Hi2OMe (30%), and the GE11-conjugated chimeric siRNA group (90%) (Supplemental Figure 13B). By day 10, the groups treated with GE11–Mseq2 Hi2OMe and GE11–Kseq2 Hi2OMe showed significant percentages of tumor growth inhibition (%TGI) of 50% and 43%, respectively, whereas the GE11-conjugated chimeric siRNA group achieved a TGI of 66% (Figure 8B). Overall, the chimeric siRNA formulation was substantially more effective at controlling tumors than either KRAS- or MYC-targeting strategy alone. When we repeated the in vivo therapeutic efficacy experiment using the A427 lung model, similar results were observed (Figure 8C and Supplemental Figure 13C). Seven days after the start of treatment, 100% of tumors in the GE11-conjugated chimeric siRNA group demonstrated reduced tumor volumes (Figure 8D). By day 18, the GE11-conjugated chimeric siRNA group achieved a TGI of 124%, whereas the groups treated with GE11–Mseq2 Hi2OMe and GE11–Kseq2 Hi2OMe showed TGIs of 55% and 39%, respectively. To evaluate the pharmacodynamic and pharmacokinetic properties of our ligand-conjugated designs, we harvested tumors 21 days after starting treatment and observed significantly diminished tumor masses in the GE11-conjugated chimeric siRNA group (Figure 8E). Using stem-loop RT-qPCR to detect individual guide strands, we confirmed that the GE11-mediated delivery platform delivered MYC and KRAS siRNAs. We observed a similar increase in the abundance of both MYC and KRAS guide strands in the GE11-conjugated chimeric siRNA–treated samples as previously seen in the H727 model, further suggesting that the chimeric design resists plasma degradation and has improved metabolic stability in the target tissue (Figure 8F). Consistent with these findings, both *MYC* and *KRAS* mRNA levels were significantly more downregulated in the GE11-conjugated chimeric siRNA group compared with the single-siRNA treatment groups (Figure 8G).

To evaluate whether the chimeric design is an improvement over coadministration of the GE11–MYC Hi2OMe and GE11–KRAS Hi2OMe individual siRNAs, we compared these treatments using the HPAF-II (KRAS^G12D^/WT) pancreatic model once tumors reached a larger size (~300 mm^3^). While mice in both treatment groups had significant tumor growth inhibition by day 7 of treatment, at day 14 the relative tumor volume in the coadministered siRNA group had returned to that of the control tumors. However, mice in the MYC/KRAS chimeric siRNA–treated group continued to demonstrate significant tumor growth inhibition (Supplemental Figure 13D). Furthermore, 60% of the mice in the MYC/KRAS chimeric siRNA treatment group survived beyond 18 days in comparison with 20% of the mice in the coadministered single-targeting MYC and KRAS siRNA group (Supplemental Figure 13E). These data show the superior activity of the MYC/KRAS chimeric siRNA formulation, which is likely more effective than coadministration of each single-targeting siRNA because of (a) its consistent uptake and targeting of both transcripts into each tumor cell (a pattern reflected in initial in vitro experiments; Figure 6B), (b) its improved potency via the additional 5′-dT overhangs (Figure 3C), and (c) its increased metabolic stability within the tumor (Figure 7B and Figure 8F).

Clinical resistance to KRAS inhibitors is well documented, with MAPK effector upregulation, MYC amplification, and YAP/TAZ signaling emerging as key players in driving resistance (67, 68). Thus, we evaluated whether the MYC/KRAS chimeric siRNA could overcome MYC amplification–driven resistance to the pan-RAS inhibitor RMC-7977 (69). Using RMC-7977 inhibitor–resistant KPC cell lines, we conducted dose-response assays. Like our observations in MYC-dependent small-cell lung carcinoma lines (Supplemental Figure 12), KRAS silencing had no impact, consistent with RAS inhibitor resistance. However, MYC siRNAs and notably MYC/KRAS chimeric siRNAs significantly inhibited all 3 resistant KPC cell lines (Supplemental Figure 14A), suggesting a further therapeutic advantage of deeper MYC silencing by dual KRAS and MYC inhibition. To assess the preliminary efficacy and safety of this approach, we evaluated whether MYC amplification–mediated resistance to pan-RAS inhibitors could be targeted in vivo. Upon tumor establishment with K18399R in C57BL/6J immunocompetent mice, mice were treated with subcutaneous treatments of GE11–negative control or GE11–M2/K2 Inverted Chimera V2 for 3 weeks (6 total doses). Compared with the control group, treatment with MYC/KRAS chimeric siRNAs significantly reduced tumor burden, with 6 mice showing complete tumor regression by day 10; however, resistance did develop in several tumors (Supplemental Figure 14, B and C). We did not observe significant changes in animal behavior, body weight, or liver or kidney function. Analysis of the complete cell differential also did not show any evidence of marrow toxicity or a systemic inflammatory response (Supplemental Figure 14, D–H).

**Figure 9:**
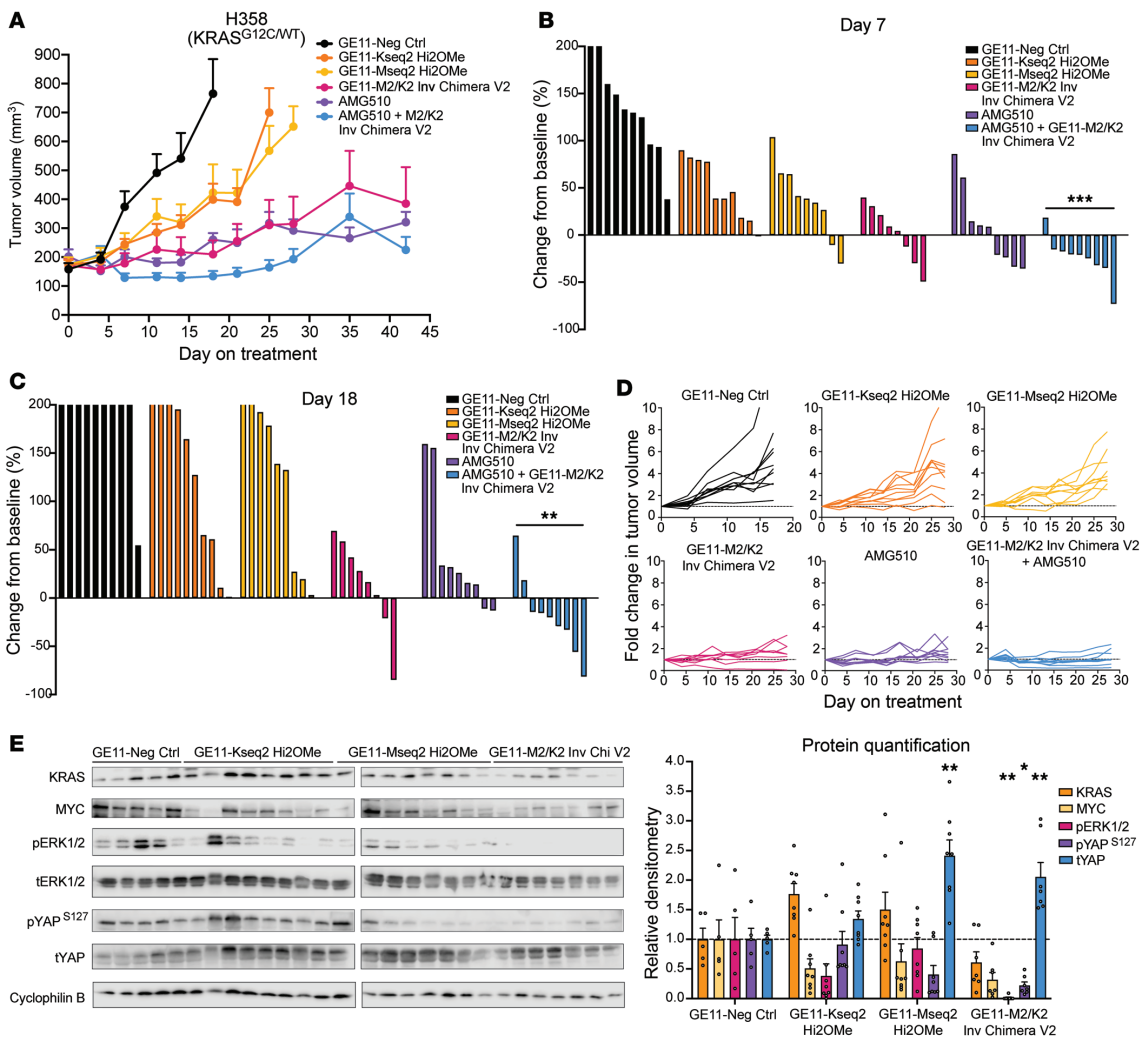
Long-term in vivo efficacy of M2/K2 inverted chimeric siRNA. (**A**) Tumor growth curves showing average fold change in H358 tumor volume over 42 days (*n* = 7–10 for all treatment groups). After 28 days, measurements were taken weekly. Error bars represent SEM. (**B**) Percentage change in H358 tumor volume for each mouse from baseline after 7 days of siRNA treatment. (**C**) Percentage change in H358 tumor volume for each mouse from baseline after 18 days of siRNA treatment. (**B** and **C**) Two-tailed Fisher’s exact test corrected for multiple hypothesis testing using the Bonferroni method was used for statistical comparisons. **P < 0.01, ***P < 0.001. (**D**) Spider plots of fold changes in H358 tumor volume for every mouse in each treatment group over 28 days. (**E**) KRAS, MYC, phospho-ERK1/2, total ERK1/2, phospho-YAP^S127^, and total YAP by Western blot in H358 tumors following treatment with GE11-conjugated negative control siRNA, MYC Hi2OMe, KRAS Hi2OMe, and M2/K2 Inverted Chimera V2. Tumors are ordered by responsiveness to treatment within each group, with strong responders at the beginning and resistant tumors at the end. Band intensities were quantified with Image Lab, (Bio-Rad) and relative band intensities (graph to the right) were calculated in comparison with negative control siRNA–treated tumors after normalization to cyclophilin B. Error bars represent SEM. Two-tailed Mann-Whitney test corrected for multiple comparisons using the Bonferroni method was used for statistical comparisons. *P < 0.05, **P < 0.01.

Finally, we compared the efficacy of the MYC/KRAS chimeric siRNA with a clinically approved KRAS^G12C^ inhibitor, sotorasib. Using the H358 (KRAS^G12C^/WT) lung adenocarcinoma model, we treated mice once subcutaneous tumors reached about 200 mm^3^ with either siRNA designs, 10 mg/kg of sotorasib, or a combination of both. Similarly to previous experiments, we observed in the H358 model that although the tumors treated with single-targeting KRAS and MYC siRNAs showed disease control in comparison with the control-treated group within a week, the rate of tumor growth was much more effectively inhibited following treatment with the MYC/KRAS chimeric siRNA (Figure 9A and Supplemental Figure 15A). By day 18, the GE11–M2/K2 Inverted Chimera V2–treated group achieved a TGI of 75%, while the groups treated with GE11–Mseq2 Hi2OMe and GE11–Kseq2 Hi2OMe showed TGIs of 50% and 52%, respectively (Figure 9B). Tumors treated with sotorasib achieved a TGI of 74% with no significant difference from those treated with the chimeric siRNA; however, a combination treatment strategy of sotorasib and MYC/KRAS chimeric siRNA administration led to highly significant responses in nearly every tumor with a peak TGI of 132%. This impressive depth of response and TGI was sustained in this group through day 21 (Figure 9C and Supplemental Figure 15B). These data suggest that targeting KRAS and downstream effectors on both a protein and an mRNA level can improve tumor burden and overall survival for a more extended period, highlighting a potential combination approach of targeting mutant KRAS protein as well as *KRAS* and *MYC* mRNA.

Knowledge about the mechanisms of resistance to KRAS inhibition is rapidly developing; however, resistance to dual KRAS and MYC inhibition is poorly understood. In the H358 model, while tumors in the chimeric siRNA group showed significant responses to treatment (and even one complete regression), many tumors eventually lost responsiveness (Figure 9D). We isolated tumors from each siRNA group and probed using Western blotting for known mechanisms of KRAS inhibitor resistance to understand whether similar pathways were responsible for driving chimeric resistance. In the individual Kseq2 treatment group, KRAS protein increased (although MYC and phospho-ERK expression remained relatively low), which complements previous research that shows increased KRAS expression as a mechanism of resistance to KRAS inhibitors (Figure 9E) (12, 16, 70). In contrast, tumors treated with the chimeric siRNA group showed maintained suppression of KRAS (30% reduction) and reductions in MYC and phospho-ERK (68% and 98% reduction, respectively), suggesting an alternative pathway of resistance. We additionally probed for phospho-YAP at serine 127, which is a marker of cytoplasmic retention of YAP, and total YAP. Several published studies have shown that activation of YAP/TAZ signaling can drive resistance to KRAS^G12C^ inhibition (71, 72). We observed a significant decrease of phospho-YAP^S127^ in the chimeric siRNA group, indicating YAP nuclear translocation and transcriptional activity. Further, we observed a significant increase in total YAP in both the individual MYC siRNA group and the chimeric siRNA group. Together these findings provide strong evidence that YAP signaling may be upregulated as a mechanism of resistance in response to dual KRAS/MYC suppression and warrant further investigation to explore potential combination therapies.

## Discussion

Therapies in oncology such as small-molecule inhibitors have resulted in remarkable improvements in survival. However, many well-characterized oncoproteins, notably MYC and about 90% of KRAS mutants, still fall into the class of “difficult-to-drug” targets. Additionally, despite the clinical success of KRAS^G12C^ inhibitors (10, 11), numerous mechanisms of primary and adaptive resistance have emerged (12–17), leading the field to consider combinatorial strategies to maximize efficacy (73). Because mutant KRAS signaling has a pivotal role in promoting downstream MYC activation through multiple mechanisms (39, 40), the ability to cotarget both oncogenes within the same cell with a single molecule represents a highly attractive drug candidate.

In this study, we developed an inverted chimeric RNAi molecule that resulted in highly potent and synergistic cotargeting of KRAS and MYC. Our results demonstrate that the guide strand of a long chimeric siRNA strand was more potent than the same guide strand delivered as a traditional single siRNA. Unexpectedly, our data support a model that this greatly enhanced potency is the result of metabolism of the chimera’s thymidine bridge, which results in deoxythymidine (dT) overhangs on the 5′ end of the KRAS guide strand. While previous studies have found that 3′-dT overhangs can affect a siRNA’s potency and in vivo stability (74), no studies to our knowledge have evaluated the effects of 5′-dT overhangs of various lengths. However, the importance of the 5′ end of the guide strand is well documented: phosphorylation of the 5′ nucleoside allows for the formation of the active RISC-siRNA complex (75), and conserving the integrity of the 5′ end is functionally more important than the 3′ end (76). During RISC loading, low base-pairing stability on the 5′ end of the guide strand characterizes siRNAs in cultured cells (77), which can also be a factor contributing to strand bias. Strategic mismatches on the 5′ end can destabilize the guide strand, leading to increased retention within the Ago complex (78, 79). We posit that the mechanistic basis for the observed increase in potency may be due to strand instability introduced by a mismatched 5′-2dT overhang on the 5′ end of the guide strand, which may reflect increased metabolic stability and could be an important factor in enhancing RNAi activity (57). This pattern of 5′-dT overhangs may be generalized to future siRNA therapeutics and should be further studied as a convenient method for increasing RNAi potency.

The modularity of the inverted chimeric siRNAs shown in this study may provide a meaningful clinical advantage over traditional strategies such as small-molecule inhibitors because of their ability to target multiple “undruggable” genes. The thymidine bridge properties of these chimeric siRNAs ensure that the individual siRNA molecules of choice are delivered to the cell in equimolar proportions, another strong advantage over single-agent small-molecule inhibitors, which can only interact with one target. Additionally, the prodrug-like metabolic processing of these chimeric molecules in acidified lysosomes leads to dramatically more potent siRNA products. We observed that the MYC/KRAS chimeric siRNA combined with an approved KRAS^G12C^ small-molecule inhibitor led to highly significant and durable reductions in tumor size, including some complete regressions, suggesting that combination approaches that cotarget KRAS at the mRNA and protein levels may be advantageous. Additionally, we observed that the MYC/KRAS chimeric siRNAs can overcome resistance to pan-RAS inhibitors (RMC-7977) that occurs through MYC amplifications, which may also have clinical implications.

Despite potential low receptor density and intratumoral heterogeneity challenges in delivering ligand-conjugated siRNAs to tumors (51), our work with an EGFR-targeting moiety demonstrates the ability to conjugate and deliver 2 linked siRNAs with a single ligand (50). The marked tumor inhibition upon chimeric siRNA treatment suggests that targeting multiple oncogenic pathways can greatly improve efficacy over a single-targeting agent. The chimeric siRNA showed strong on-target suppression of KRAS and MYC in tumors, and preliminary toxicology studies indicate that this modality may be safe. However, more extensive safety studies will be needed before it enters the clinic, notably regarding its impact in other highly EGFR-expressing tissues (such as skin and the bladder) and in the kidney, which is the main site of clearance for ligand-conjugated siRNAs. To demonstrate long-term safety, additional studies including dose escalation experiments to establish toxicity limits, expanded transcriptional profiling for off-target effects, and validation in additional animal models will need to be performed.

Finally, our efficacy experiments in larger tumors indicate that there may be limitations to achieving sufficient delivery of this RNAi molecule, which may be related to increased tumor heterogeneity, disrupted vascular perfusion, or insufficient tumor loading, and should be further investigated. Additionally, despite the potential increase in therapeutic window under the chimeric siRNA, continued treatment does show the eventual development of resistance through YAP signaling, which may be a driver of resistance under dual KRAS/MYC protein suppression. Further optimization of ligand-conjugated delivery of inverted chimeras may enable improved selectivity and potency of these molecules while reducing toxic side effects. The features of the inverted chimeric siRNAs are attractive and applicable to other complex diseases beyond cancer that may require dual targeting, such as cardiometabolic disorders, neurodegeneration, inflammation, or infectious diseases (80).

## Methods

Further information can be found in Supplemental Methods.

### Sex as a biological variable.

For murine studies, 8- to 12-week-old female athymic nude or C57BL/6 mice were used. Sex was not considered as a biological variable, as the incidence and outcome of human lung and pancreatic cancers are nearly equivalent for each sex.

### siRNA transfections.

The sequences of all siRNAs are in Supplemental Table 1 and as previously described (54). All siRNA transfection experiments were completed using Lipofectamine RNAiMAX (Life Technologies) in culture medium without antibiotics following manufacturer instructions.

### RT-qPCR.

Total RNA from cell lysates was purified using the Quick RNA MicroPrep Zymo Research Kit (Genesee Scientific). For mRNA analysis, cDNA was synthesized using the iScript cDNA Synthesis Kit (Bio-Rad) per the manufacturer’s instructions. Analysis of RNA levels was determined by a StepOnePlus Real-Time PCR System (Applied Biosystems) using SYBR Green Master Mix (Bio-Rad). A list of gene-specific primers used for RT-qPCR is shown in Supplemental Table 3. Reactions were run in duplicate or triplicate. Fold change was calculated using the 2^–ΔΔCt^ method, and experiments were normalized to expression of the rRNA 18S and expression of target genes in the negative control–treated samples. Graphs were generated with GraphPad Prism.

### Cell viability experiments.

Cell viability in response to siRNA treatment was evaluated with the CellTiter-Glo 2.0 Cell Viability Assay using the manufacturer’s protocol (Promega). Resuspended MIA PaCa-2 cells in culture medium were seeded at 1,000 cells per well, and resuspended A427 cells were seeded at 3,500 cells per well, in opaque, flat-bottom 96-well plates. All cells were counted with the Countess 3 Automated Cell Counter (Thermo Fisher Scientific). All siRNAs (suspended in serum-free medium with Lipofectamine RNAiMAX) were tested in triplicate starting at 40 or 20 nM and progressing through a 10-point serial dilution. Plates were incubated in culture conditions for 5–6 days. For viability readouts, 120 μL of medium was removed from each well, and an equal volume of CellTiter-Glo 2.0 (CTG) Reagent was added. Luminescence was measured at 530 nm excitation and 590 nm emission on a Synergy2 fluorescent plate reader (BioTek). Data were analyzed in GraphPad Prism. Experiments were repeated at least 3 times, and representative dose-response curves are shown in the figures.

### Luciferase experiments.

Changes in KRAS–firefly luciferase expression in response to siRNA treatment were evaluated with the Luc-Pair Duo-Luciferase HT Assay Kit using the manufacturer’s protocol (Genecopoeia). Resuspended cells in culture medium were added to opaque, flat-bottom 96-well plates. A-431 KRAS-luciferase cells were seeded at 3,500 cells per well and were counted with the Countess 3 Automated Cell Counter (Thermo Fisher Scientific). All siRNAs (suspended in serum-free medium with Lipofectamine RNAiMAX) were tested in triplicate starting at 40 or 20 nM and progressing through a 10-point serial dilution. Plates were incubated in culture conditions for 3–4 days. For luciferase readouts, 120 μL of medium was removed from each well, and an equal volume of working FLuc reagent was added and incubated for 10 minutes. Luminescence was measured at 530 nm excitation and 590 nm emission on a Synergy2 fluorescent plate reader (BioTek). An equal volume of working RLuc reagent was subsequently added and incubated for an additional 5 minutes, and luminescence was measured as above. The ratio of luminescence from the firefly luciferase to the Renilla luciferase was then calculated. Data were analyzed in GraphPad Prism, and ED_50_ curves were produced. Relative potency was calculated by division of the ED_50_ value of the Kseq2 Hi2Ome–treated cells by the ED_50_ value of the other conditions.

### 3D spheroid formation assay.

A427 and MIA PaCa-2 cells were seeded into 12-well plates and treated with 5, 10, or 20 nM of siRNAs and Lipofectamine RNAiMax in culture medium without antibiotic for 24 hours. Cells were then lifted with trypsin and counted. Five thousand cells from each condition were mixed with 50 μL of cold Matrigel (Corning) and plated onto 24-well glass-bottom plates. After solidification of the matrix, complete medium with 10% FBS and antibiotic was added to every well. Plates were incubated for 4–5 days and then imaged with a Leica Dmi8 inverted microscope (×5 objective). Spheroid area and number in each condition were quantified using Organoseg software (81). Graphs were generated using GraphPad Prism.

### In vivo modeling and tissue processing.

Animals were cared for according to guidelines set forth by the Association for Assessment and Accreditation of Laboratory Animal Care International and the US Public Health Service Policy on Humane Care and Use of Laboratory Animals. Mouse studies were approved and supervised by the University of North Carolina at Chapel Hill Institutional Animal Care and Use Committee. Athymic nude mice or C57BL/6 mice were between 8 and 12 weeks of age at the time of injection. Cells were trypsinized, washed, and resuspended in Hanks balanced salt solution (HBSS; Gibco), and 3.5 × 10^6^ A427, H358, H727, or HPAF-II cells or 5 × 10^5^ K18399R cells were injected subcutaneously in a 50 μL 1:1 mixture of HBSS and BD Matrigel (BD Biosciences). Caliper measurements of subcutaneous tumor growth were taken twice weekly (unless otherwise indicated), and tumor volume was calculated as *L* × *W*^2^ where *L* is the greatest cross-sectional length across the tumor and *W* is the length perpendicular to *L*. Once tumors reached about 75–300 mm^3^ in volume, mice were randomly assigned to treatment groups and injected subcutaneously twice weekly at either 5 mg/kg for a single-targeting siRNA, 10 mg/kg (thus 5 mg/kg of each siRNA) for the chimeras, or 10 mg/kg of sotorasib. Peptides were synthesized by the Chemical Products Corporation and sent to Avecia or Synoligo for conjugation to modified siRNAs. Sotorasib (AMG510) was purchased from MedKoo (CAS number 2252403-46-6). Tumor weights and blood were obtained after necropsy, and tumors were snap-frozen or fixed in 10% formalin before downstream analyses.

### Statistics.

Results for each group were compared using unpaired 1-tailed Student’s *t* test corrected for multiple comparisons using the Bonferroni method, Mann-Whitney test corrected for multiple comparisons using the Bonferroni method (if the data did not have a Gaussian distribution), 1-tailed analysis of variance (ANOVA), or Fisher’s exact test (for contingency analysis). For survival studies, log-rank (Mantel-Cox) test was used. A *P* value less than 0.05 was deemed statistically significant unless otherwise stated in the figure legend. All statistical tests for in vitro and in vivo experiments were performed using Prism 7 (GraphPad Software Inc.).

### Study approval.

Mouse studies were approved and supervised by the University of North Carolina at Chapel Hill Institutional Animal Care and Use Committee.

### Data availability.

All data generated or analyzed during this study (including values for all data points) are included in this published article and its supplemental information and Supporting Data Values file. Sequencing data can be accessed with Gene Expression Omnibus (GEO) accession number GSE261735. Any unique biological materials are available upon request.

## Author contributions

YSC, AAB, ME, and CVP were involved in conceptualization of the project and relevant aims. AP was involved in data curation and software implementation for large datasets (including RNA-Seq data). YSC, HPH, SSS, LJS, CGF, KMW, LE, SHA, MCF, JI, AP, JLP, and IS were involved in formal analysis, investigation, methodology, validation, and visualization for the data included in the article. CVP is the primary project administrator and acquired funding for the project. All authors read, edited, and approved the final manuscript.

## Supplementary Material

Supplemental data

Unedited blot and gel images

Supplemental table 1

Supplemental table 2

Supplemental table 3

Supporting data values

## Version 1 07/31/2025

In-Press Preview

## Version 2 10/01/2025

Electronic publication
