# Supplementary material for: Inverted chimeric RNAi molecules synergistically cotarget MYC and KRAS in KRAS-driven cancers
Source: J Clin Invest. 2025 Jul 22;135(19):e187204. doi: 10.1172/JCI187204 (PMC12483557; doi:10.1172/JCI187204)
Supplement: Supplemental data [file jci-135-187204-s162.pdf]

Inverted chimeric RNAi molecules synergistically co-target MYC and KRAS in KRAS-driven cancer

Yogitha S. Chareddy, Hayden P. Huggins, Snehasudha S. Sahoo, Lyla J. Stanland, Christina Gutierrez-Ford, Kristina Whately, Lincy Edatt, Salma H. Azam, Matthew C. Fleming, Jonah Im, Alessandro Porrello, Imani Simmons, Jillian L. Perry, Albert A. Bowers, Martin Egli, Chad V. Pecot

Supplemental Methods

siRNA design

The full human and mouse *c-Myc* (*MYC*) sequences (CCDS6359.2 and 27504.1) were used as input for several open-source siRNA design websites, including siDesign Center, BLOCK-iT Designer, IDT DsiRNA, and siRNA Target Finder. The top hits from each tool for human *MYC* were compared to find sequences that were suggested multiple times and then subsequently compared for cross-reactivity against mouse *MYC*. The final list of siRNAs for testing contained sequences that could target both human and mouse *MYC* (Supplemental Table 1). siRNAs were synthesized by Sigma-Aldrich.

Cells and culture conditions

Most cell lines were obtained from the ATCC (unless otherwise indicated) and routinely tested for mycoplasma using a Lonza MycoAlert Detection kit (LT07-418). All culture media contained 10% Fetal Bovine Serum (FBS) (Avantor) and 1% Penicillin–Streptomycin (P-S) (Sigma) antibiotic. MIA PaCa-2 cells, HEK293T Parental and NoDice cells (kindly obtained from the Cullen lab) (1), K18399R, K18745R, and K18509R (kindly obtained from Ken Olive's lab) (2) and A-431 KRAS-knockout cells (3) were grown in Dulbecco's Modified Eagle's Medium (DMEM) media (Gibco). A-431 KRAS-knockout cells were transduced with lentiviral constructs expressing KRAS:firefly luciferase/renilla luciferase (Genecopoeia) and then selected and maintained in puromycin (1 µg/ml). A427 cells were grown in Modified Eagle's Medium (MEM) media (Gibco). LU65 (kindly obtained from the Hata lab), NCI-H358, and NCI-H441 cells were grown in RPMI-1640 media (Gibco). HCT116 cells were grown in McCoy's 5A medium (Gibco). HPAF-II cells were grown in Eagle's Minimum Essential Medium (EMEM) (ATCC). SW 1271 cells were grown in Leibovitz's L-15 medium (Gibco). H1048 and H841 cells were grown in DMEM/F12 medium (Gibco) supplemented with the following: 0.005 mg/ml insulin, 0.01 mg/ml transferrin, 30nM sodium

selenite (final conc.), 10 nM hydrocortisone (final conc.), 10 nM beta-estradiol (final conc.), and extra 2mM L-glutamine (for final conc. of 4.5 mM). All cell lines except for SW 1271 cells were grown in T75 flasks at 37°C with 5% CO_2/95% air. SW 1271 cells were grown in a T75 flask with a closed plug seal cap at 37°C with 5% CO_2/95% air to prevent oxygen exchange.

Western blotting

RIPA buffer (Thermo) containing complete protease inhibitor cocktail (Roche) and Halt phosphatase inhibitor cocktail (Thermo) was used to lyse cells following treatment. Total protein was mixed with 4X Laemmli buffer and 5% 2-Mercaptoethanol, denatured at 95°C for 5 min., and loaded onto 10% SDS-PAGE gels. Protein was transferred to nitrocellulose membranes (Bio-Rad) overnight. 5% bovine serum albumin, 5% non-fat dried milk, or SuperBlock Blocking Buffer (Thermo Fisher) (according to manufacturer protocol for each primary antibody) in tris-buffered saline with tween-20 (TBS-T) was used to block membranes for one hour at room temperature. Primary antibody diluted 1:1000 or 1:500 was used to probe the membrane overnight at 4°C or one hour at room temperature (according to manufacturer protocol). Primary antibodies included anti-MYC (D84C12, #5605) from Cell Signaling Technology, anti-vinculin (clone hVIN-1, #V9131) from Sigma, anti-KRAS from Sigma (lot J7111-S2, #WH0003845M1), P-p44/42 MAPK (pERK1/2) from Cell Signaling Technology (#4370), p44/42 MAPK (ERK1/2) from Cell Signaling Technology (#9102), P-S6 from Cell Signaling Technology (#2211), YAP from Cell Signaling Technology (#4912) and Phospho-YAP (Ser127) from Cell Signaling Technology (#4911). Membranes were then washed three times with TBS-T and re-probed with the appropriate horseradish peroxidase-conjugated secondary antibodies (anti-mouse (#115-035-003) or anti-rabbit (#111-035-003) from Jackson ImmunoResearch) for one hour at room temperature. Membranes were then washed three times in TBS-T and developed using Clarity Western ECL substrate (Bio-Rad) or SuperSignal West Femto (Thermo), visualized with a Bio-Rad ChemiDoc MP system (Bio-Rad). Band intensities were quantified with Image Lab. Relative band intensities were calculated in

comparison to negative control siRNA-treated cells at the comparative dose and expression of each protein were normalized as follows: KRAS, phospho-S6, and MYC were normalized to the loading control (either vinculin or cyclophilin B), and phospho-ERK1/2 and phospho-YAP^{S127} were normalized to the loading controls and then their respective total protein levels. For quantification, each protein of interest was normalized to its corresponding loading control measurements, although only the most representative loading control images were selected for figure presentation.

Stability assays

10µM of siRNAs was incubated in 50% Fetal Bovine Serum (FBS) in sterile PBS at 37°C. At the end of each time point, 0.5M EDTA was added to quench nuclease activity, and samples were placed at -80°C. *Rat Liver Tritosomes*. Rat liver tritosomes (Xenotech) were diluted to 0.5 mg/ml in 20mM sodium citrate, pH 5.0. 4µM of siRNAs were mixed with acidified tritosomes and incubated at 37°C. At the end of each timepoint, 0.5M EDTA was added and samples were placed at -80°C. *Rat Liver Cytosol.* 10µM of siRNAs was incubated in 105mM phosphate buffer with 2mg/ml rat liver cytosol (Xenotech) at 37°C. At the end of each timepoint, 0.5M EDTA was added and samples were placed at -80°C. 1X loading dye was added to all samples prior to electrophoresis. All samples for stability assays were run on a 16% non-denaturing polyacrylamide gel for 90-120 min at 75V in 1X TBE buffer. The gel was stained with SYBR Safe following manufacturer's protocol and visualized with a Bio-Rad ChemiDoc MP system (Bio-Rad). Band intensities were quantified with ImageJ.

Liquid chromatography–mass spectrometry

Rat liver tritosomes (Xenotech) were diluted to 0.5 mg/ml in 20mM sodium citrate, pH 5.0. 20µM of siRNAs were mixed with acidified tritosomes and incubated at 37°C. At the end of each timepoint, 0.5M EDTA was added and samples were placed at -80°C. Once all samples were

collected, they were treated with 1% SDS and boiled for 10 min before being processed through a 25 mg/well plate in the Clarity OTX kit (Phenomenex). The optimized manufacturer's protocol was adapted with the following changes: instead of a vacuum manifold, a plate centrifuge was used at 300 rcf for 30 seconds per step. Prior to elution, excess wash buffer was removed by a single 800 rcf spin for 30 seconds. Following elution, samples were lyophilized in glass vials and resuspended in water. Samples were analyzed on a Waters Acquity UPLC coupled to a Orbitrap fusion in negative ion mode (Thermo Fisher Scientific). LC-UV 260 nm data was also collected with the LC-MS data. The data was processed using ProMass software (Novatia, LLC) with its Positive Probability Ltd (PPL) deisotoping algorithm (licensed to Novatia, LLC) and a 0.5% spectrum labeling threshold.

In vitro Dicer treatment

10µM of siRNAs was incubated at 37°C with 1600 fmols of recombinant human Dicer (purchased from Origene, cat #TP319214) in standard Dicer reaction buffer (250mM NaCl, 20mM Tris-HCl pH 7.5, and 2.5mM $MgCl_2$).

Modeling of chimeric siRNA construct and siRNA:Ago2 complexes

To build the inverted chimeric MYC-KRAS siRNA duplex, we used the 3DNA web server (https://x3dna.org/) (4) to generate a self-complementary A-form duplex based on fiber diffraction parameters for helical rise and twist. All further manipulations of the initial model were done in the UCSF Chimera suite (5). Overhangs at the 3'-ends of guide strands were created by deleting residues on the pairing strand, the single-stranded $d(T)_4$ linker was produced by deleting adenosines on the opposite strand and converting U to dT, 5'-terminal phosphates were retained as needed, and phosphorothioate linkages were arbitrarily treated as either of the *R*p- or *S*p-configuration. All ribonucleotides were either converted to 2'-*O*-methyl or 2'-F residues. To kink the straight chimeric siRNA duplex, we changed the backbone torsion angles in the $d(T)_4$ linker.

The crystal structure of human Ago2 bound to guide and target RNA containing seed pairing (6) (PDB ID 4W5T) served as the starting model to construct complexes of Ago2 in complex with either the 4dT-linked MYC passenger and KRAS guide strands or the KRAS guide strand with a 2dT overhang at the 5'-end. For both models, the conformations of the thymidine portions were manipulated manually. The position of the terminal phosphate in the MID domain binding pocket was left unchanged to optimize the arrangements of $d(T)_4$ and $d(T)_2$ wedged between MID and PIWI domain residues. Both models were energy-minimized with Amber 14 (https://ambermd.org/) (7), using a steepest-descent protocol until conversion. It is difficult to avoid clashes to weave the 4dT linker out of the binding cleft to fuse it with the MYC passenger strand, but the 5'-terminal 2dT overhang can be quite well accommodated in the modeled complex.

Bioinformatics and statistical analyses of RNA-sequencing data.

Illumina FASTQ paired-end files produced by the Illumina NovaSeq 6000 sequencer (8) were, for each sample, jointly used. The alignment to the hg38 human reference genome was performed through STAR 2.7.6a (9) (parameters: --outSAMunmapped Within --outSAMtype BAM Unsorted --quantMode TranscriptomeSAM). The quantification of transcripts was performed using Salmon 1.4.0 (10), based on the human transcriptome defined by GENCODE, Release 36(11). Then, a summary process created a cohort-wide matrix showing the expression, for all samples, of the complete set of gene IDs. Thereafter, samples were processed to remove gene IDs with lower average counts across sequenced samples, thus restricting the expression matrix to gene IDs belonging to the upper fourth decile. For each experimental comparison for A427 cells (Mseq2 Hi2OMe, Kseq2 Hi2OMe, and M2/K2 Inverted Chimera V2, each vs. negative control), we performed a differential expression analysis through DESeq2 (1.36.0) (12), using the rounded Salmon-quantified gene expression matrix, within the R (4.2) software environment. In every analysis, we collected baseMean, p-value, adjusted p-value, and log2 fold change using the

‘results’ function of DESeq2, with alpha = 0.0001. Following authors’ recommendations, we separately calculated gene fold changes using the apeglm R package (13) to produce shrinkage estimators of effect sizes. Using the apeglm log2 fold changes and standard adjusted p-values of DESeq2, we created volcano plots having, on the x-axis, log2(fold change) and on the y-axis, -log10(adjusted p-value). These plots were made using GraphPad Prism 9 (GraphPad). For the few cases (11, 1, and 1, respectively) when the adjusted p-values were below the minimum value that DESeq2 could accurately calculate, we conservatively approximated such ‘null’ values with the lowest adjusted p-value, separately for each differential expression analysis. Finally, only coding genes recognized by the Human Genome Organisation (HUGO) (14) were kept in the displayed volcano plots. GEO accession number: GSE261735 (enter token cvqrsqawljundkz).

Immunohistochemical staining

Cleaved Caspase-3 antibody was purchased from Cell Signaling (Danvers, MA, USA; catalog number: 9664S and c-Myc and Ki67 antibodies were from Cell Marque (Rocklin, CA, USA; catalog numbers: 395R-14 and 275R-14, respectively). Chromogenic immunohistochemistry (IHC) was performed on paraffin-embedded tissues that were sectioned at 5 micrometers. The IHC was carried out using the Leica Bond III Autostainer system. Slides were deparaffinized in Bond Dewax solution (AR9222) and hydrated in Bond Wash solution (AR9590). Heat induced antigen retrieval was performed for 20 min at 100^{0}C in Bond-epitope retrieval solution 2, pH 9.0 (Leica, AR9640) in detection of cleaved Caspase-3 antigen or Bond-epitope retrieval solution 1 pH 6.0 (Leica, AR9961) in detection of the remaining antigens. Nonspecific binding was blocked with Background Sniper (Biocare Medical, catalog number BS966M) at room temperature for 10 min in samples to be incubated with cleaved Caspase-3 and Ki67. After pretreatment, samples were treated with the antibody solutions: cleaved Caspase-3 at 1:500 dilution for 30 min, c-Myc at 1:50 dilution for 4 h, or Ki67 at dilution 1:200 for 1 h. Then, each sample was incubated in the Novolink Polymer secondary antibody (Leica, RE7161) for 8 min. Antibody detection with 3,3'-

diaminobenzidine (DAB) was performed using the Bond Intense R detection system (DS9263). Stained slides were dehydrated and coverslipped with Cytoseal 60 (epredia, 8310-4). The slides were imaged at 20x magnification using the Axio Observer 7 (ZEISS). Positive cells per high powered field were determined using a pipeline in CellProfiler 4.2 software.

Detection of chimeric siRNAs in xenograft tumors using stem-loop RT-qPCR

A427 or H727 xenograft tumors were collected from a cross-sectional necropsy of athymic mice treated with GE11- or HW12-conjugated siRNAs and snap-frozen in liquid nitrogen. Tumors were stored at -80°C until processed. Tumors were dissected on dry ice and RNA was isolated from 40-60 mg of tissue using Trizol® Reagent (Thermo Fisher Scientific) according to manufacturer instructions with the following alterations: tumors were lysed in 1 mL of Trizol® Reagent using a bead mill (speed: 3.7 m/s, time: 00:30 s, frequency: 3 times, incubating at 4°C for 2 min between each pulse), 30 µgs of Rnase free glycogen was added to the isolated aqueous layer as a co-precipitant, RNA was precipitated in an equal volume of isopropanol at -20°C for 20 mins, RNA pellet was washed twice in 70% EtOH, and resuspended in 100 µLs of nuclease-free water. cDNA was synthesized using the TaqMan™ MicroRNA Reverse Transcription Kit (Thermo Fisher Scientific) according to the manufacturer's instructions with the following alterations: 5 µLs of total RNA was denatured in the presence of a sequence-specific stem-loop RT primer pool in a thermocycler (85°C 5 min., 60°C 5 min., 4°C 5 min.) prior to reverse transcription for optimal primer annealing. RT-qPCR was performed in duplicate on a QuantStudio™ 3 system (Applied Biosystems) using TaqMan™ Universal Master Mix II, no UNG (Applied Biosystems), and Custom TaqMan™ Small RNA Assay probes (Applied Biosystems) specific to the siRNAs of interest, according to the manufacturer's instructions. To calculate relative guide strand abundance, all C_T values were transformed using 2^{-C_T} and then divided by the tumor mass. For KRAS abundance, these numbers were relativized to the values from the GE11-Kseq2 Hi2OMe-treated group. For

MYC abundance, these numbers were relativized to the values from the GE11-Mseq2 Hi2OMe-treated group.

Supplemental Figures

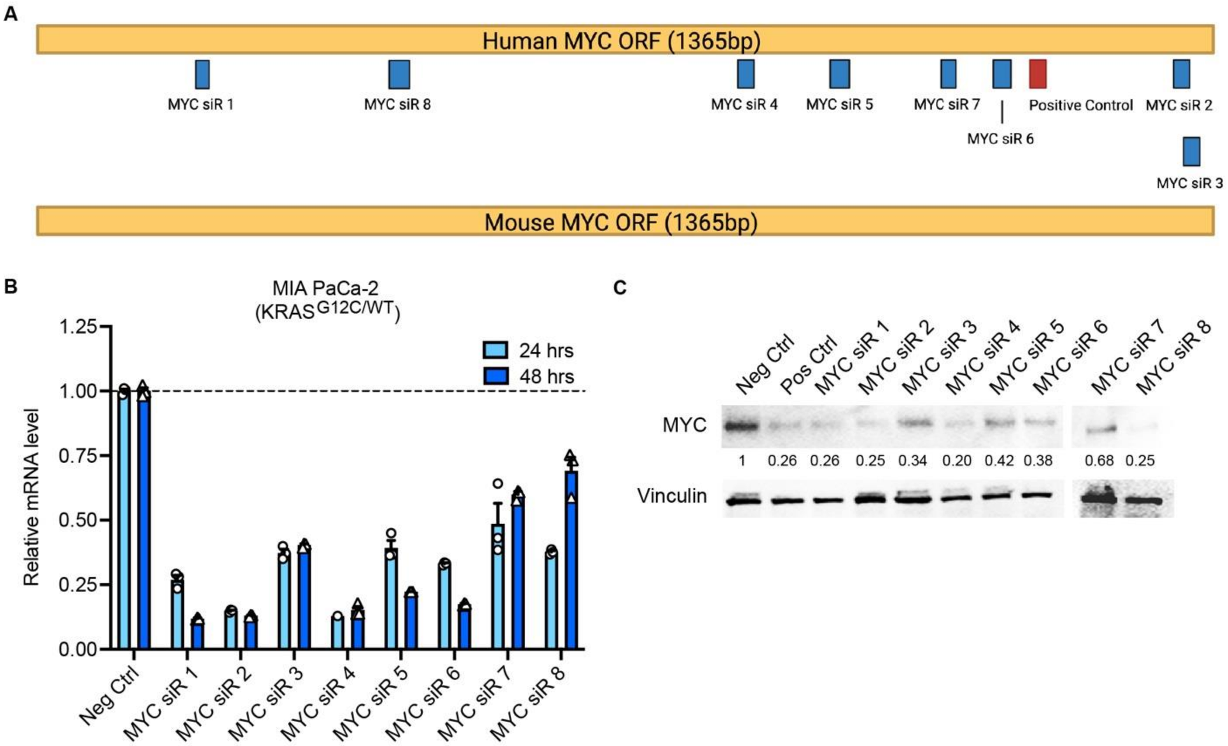

Supplemental Figure 1. Screening MYC-targeting siRNAs. (**A**) Schematic showing the human and mouse *MYC* open reading frames and the target sites for eight screened MYC-targeting siRNAs and one positive control siRNA. Created with Biorender.com. (**B**) *MYC* mRNA expression by RT-qPCR in MIA PaCa-2 cells following treatment with the non-targeting negative control and eight MYC-targeting siRNAs at 20nM for 24 and 48 hrs. Error bars represent SEM. (**C**) MYC protein expression by Western blot in MIA PaCa-2 cells following treatment with the control and eight MYC-targeting siRNAs at 20nM for 24 hrs. Band intensities were quantified with ImageJ. MYC protein expression was normalized to vinculin and relative band intensities were calculated in comparison to control siRNA-treated cells.

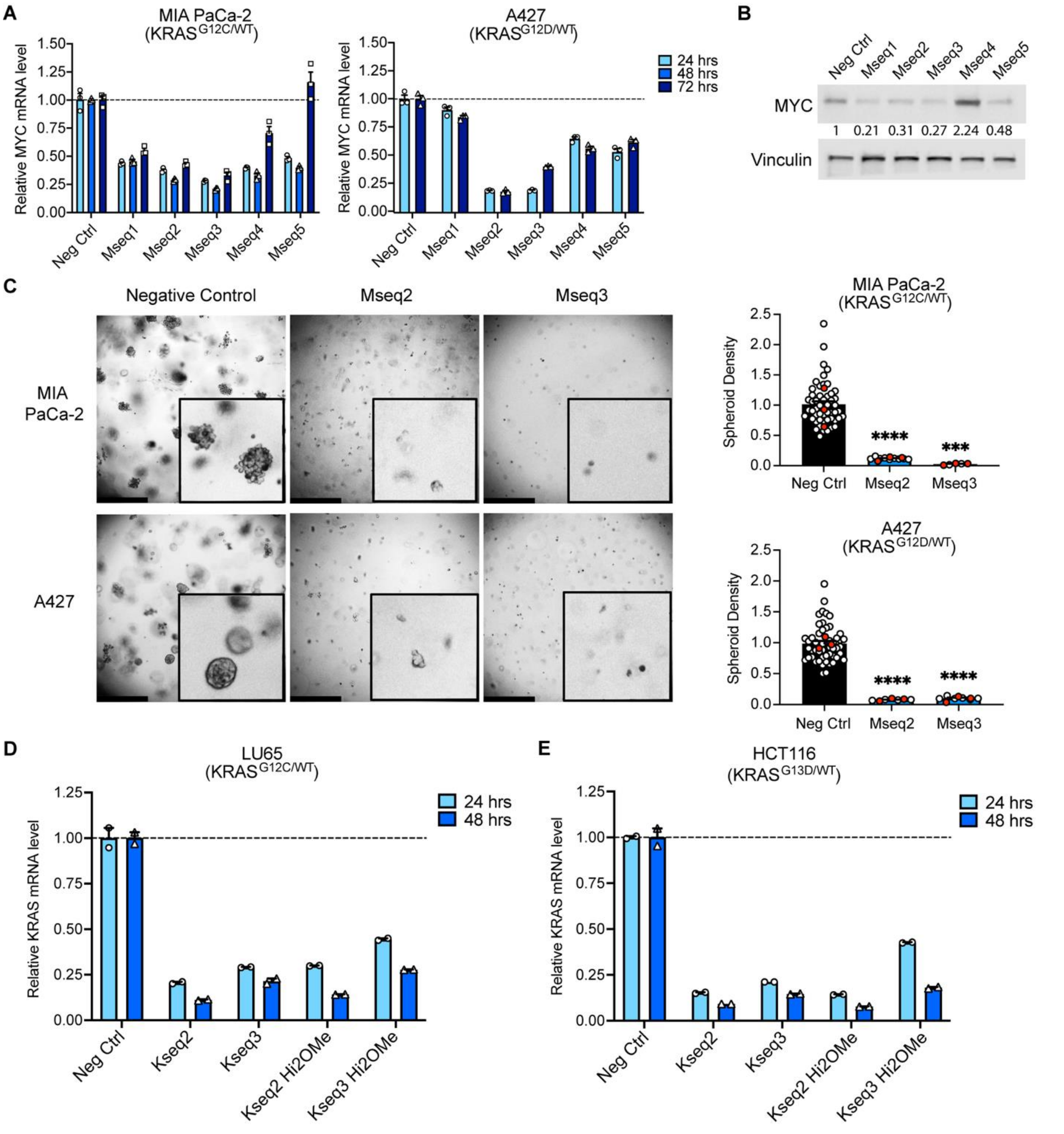

Supplemental Figure 2. In-vitro activity of chemically modified MYC and KRAS-targeting siRNAs. (**A**) Relative *MYC* expression by RT-qPCR in MIA PaCa-2 and A427 cells following treatment with the control and five modified MYC-targeting siRNAs at 20nM for 24, 48, and 72 hrs. Error bars represent SEM. (**B**) MYC expression by Western blot in MIA PaCa-2 cells following treatment with the negative control and eight MYC-targeting siRNAs at 20nM for 72 hrs. Band intensities were quantified with ImageJ. Relative band intensities were calculated in comparison

to control siRNA-treated cells and MYC expression was normalized to vinculin. (**C**) Representative images and quantification of spheroids in a tumorigenesis assay in Matrigel with A427 and MIA PaCa-2 cells. siRNA-treated cells were mixed with Matrigel and grown for five days on glass-bottom plates. Images were taken with a 5x microscope objective. Scale bar, 498mm. Average spheroid area and number were quantified by Organoseg. The average spheroid area and number from three images per condition were averaged and normalized to the control siRNA-treated cells. Error bars represent SEM. One-way ANOVA was used for statistical comparisons. (****) = $p<0.0001$, (***) = $p<0.001$. (**D-E**) Relative *MYC* expression by RT-qPCR in (**D**) LU65 and (**E**) HCT116 cells following treatment with the negative control, two unmodified KRAS-targeting siRNAs, and two Hi2OMe KRAS-targeting siRNAs at 20nM for 24 and 48 hrs. Error bars represent SEM.

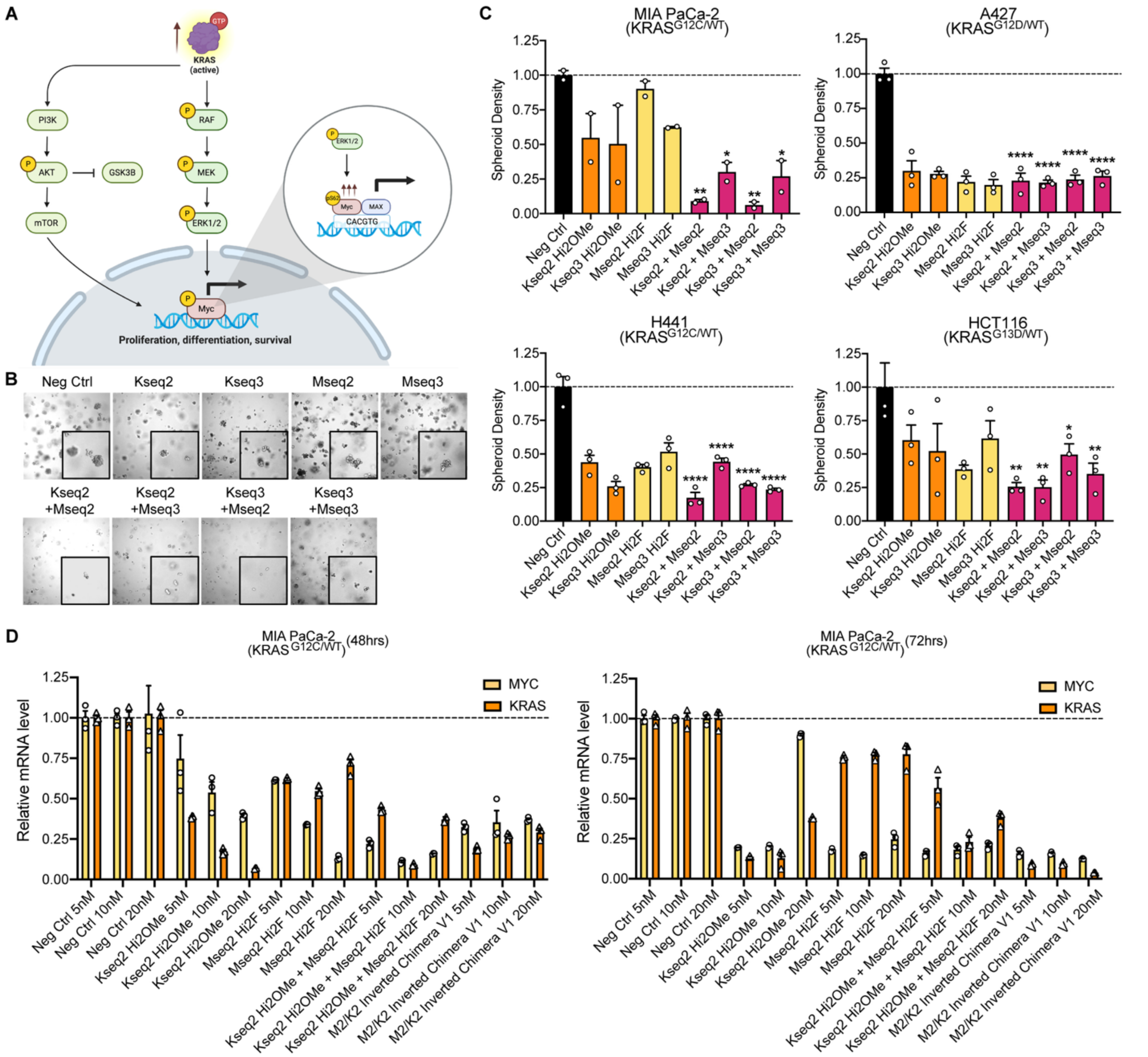

Supplemental Figure 3. Increased potency by siRNA-mediated dual-targeting of MYC and KRAS. (**A**) Schematic showing the oncogenic relationship between KRAS and MYC. Hyperactive KRAS can lead to stabilization and accumulation of active MYC, driving several pro-survival and proliferative cellular programs. Created with Biorender.com. (**B**) Representative images of spheroids in a tumorigenesis assay in Matrigel with MIA PaCa-2 cells. 5nM of each siRNA was used to treat cells that were then mixed with Matrigel and grown for five days on glass-bottom plates. The combo conditions were treated with a mixture containing 5nM of each siRNA. Images were taken with a 5x microscope objective. Scale bar, 498mm. (**C**) Quantification of spheroids in

a tumorigenesis assay in Matrigel with MIA PaCa-2, A427, H441, and HCT116 cells. 5nM of siRNAs was used to treat MIA PaCa-2, H441, and HCT116 and 10nM of siRNAs was used to treat A427. In conditions with MYC + KRAS co-transfection, each of the MYC and KRAS siRNAs were transfected at the indicated dose. Average spheroid area and number were quantified by Organoseg. The average spheroid area and number from three images per condition were averaged and normalized to the control siRNA-treated cells. Error bars represent SEM. One-way ANOVA was used for statistical comparisons. (****) = $p<0.0001$, (**) = $p<0.01$, (*) = $p<0.05$. (**D**) Relative *MYC* and *KRAS* expression by qPCR in MIA PaCa-2 cells following treatment with the negative control, MYC Hi2F, KRAS Hi2OMe, combo MYC and KRAS, and M2/K2 Inverted Chimera V1 siRNAs at 5, 10, and 20nM for 48 and 72 hrs. In conditions with MYC + KRAS co-transfection, each of the MYC and KRAS siRNAs were transfected at the indicated dose. Relative mRNA expression level was calculated in comparison to control siRNA-treated cells and normalized to 18S. Error bars represent SEM.

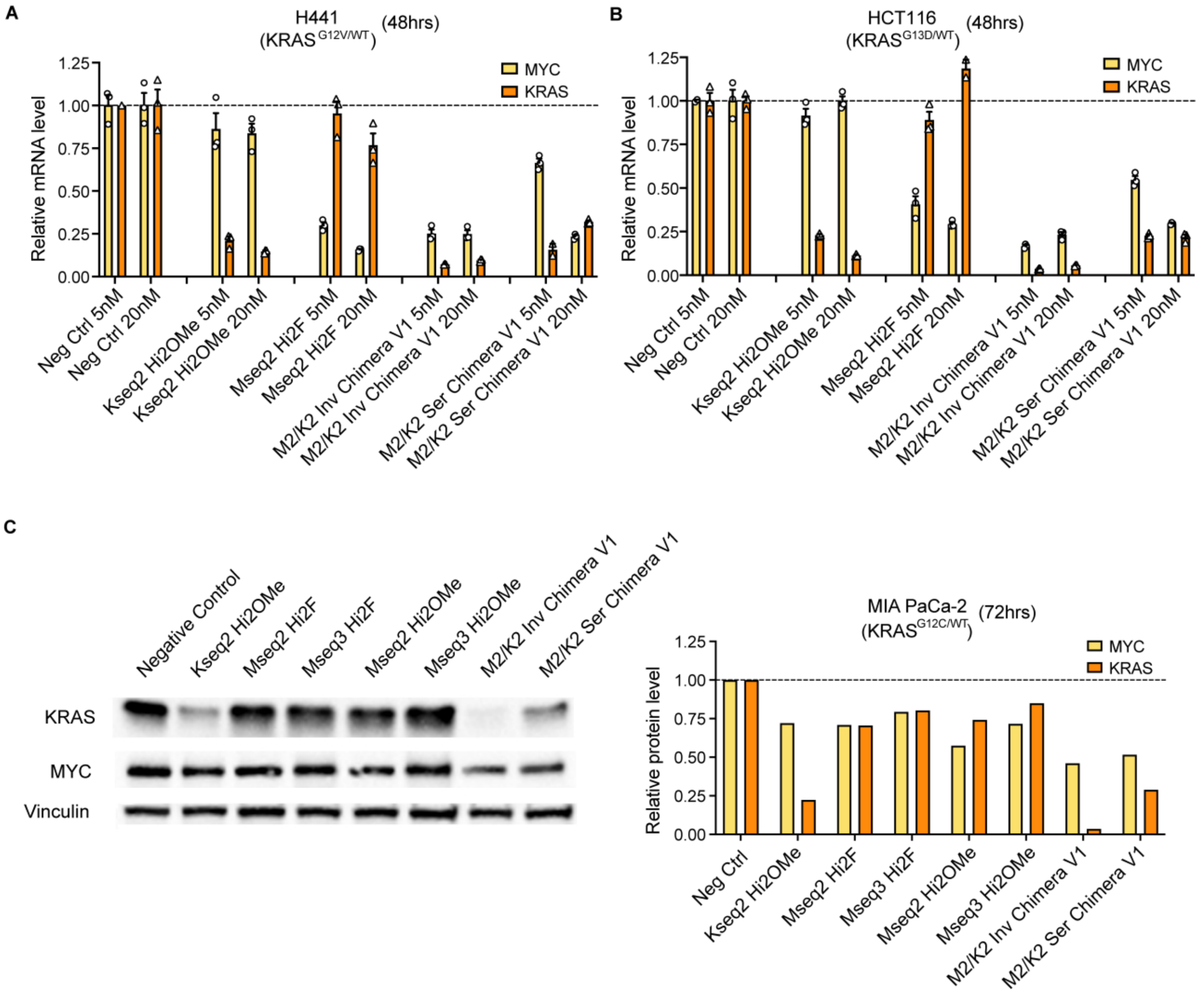

Supplemental Figure 4. Additional assays on in-vitro activity of MYC-KRAS chimeric siRNAs. (**A-B**) Relative *MYC* and *KRAS* mRNA expression by RT-qPCR after siRNA treatment at 5 and 20nM for 72 hrs in (**A**) H441 cells and (**B**) HCT116 cells. Error bars represent SEM. (**C**) MYC and KRAS expression by Western blot in MIA PaCa-2 cells following treatment with the negative control, KRAS, two MYC Hi2F, two MYC Hi2OMe, M2/K2 Inverted Chimera V1, and M2/K2 Serial Chimera V1 siRNAs at 20nM for 72 hrs. Band intensities were quantified with ImageJ. Relative band intensities were calculated in comparison to control siRNA-treated cells and MYC and KRAS expression were normalized to vinculin.

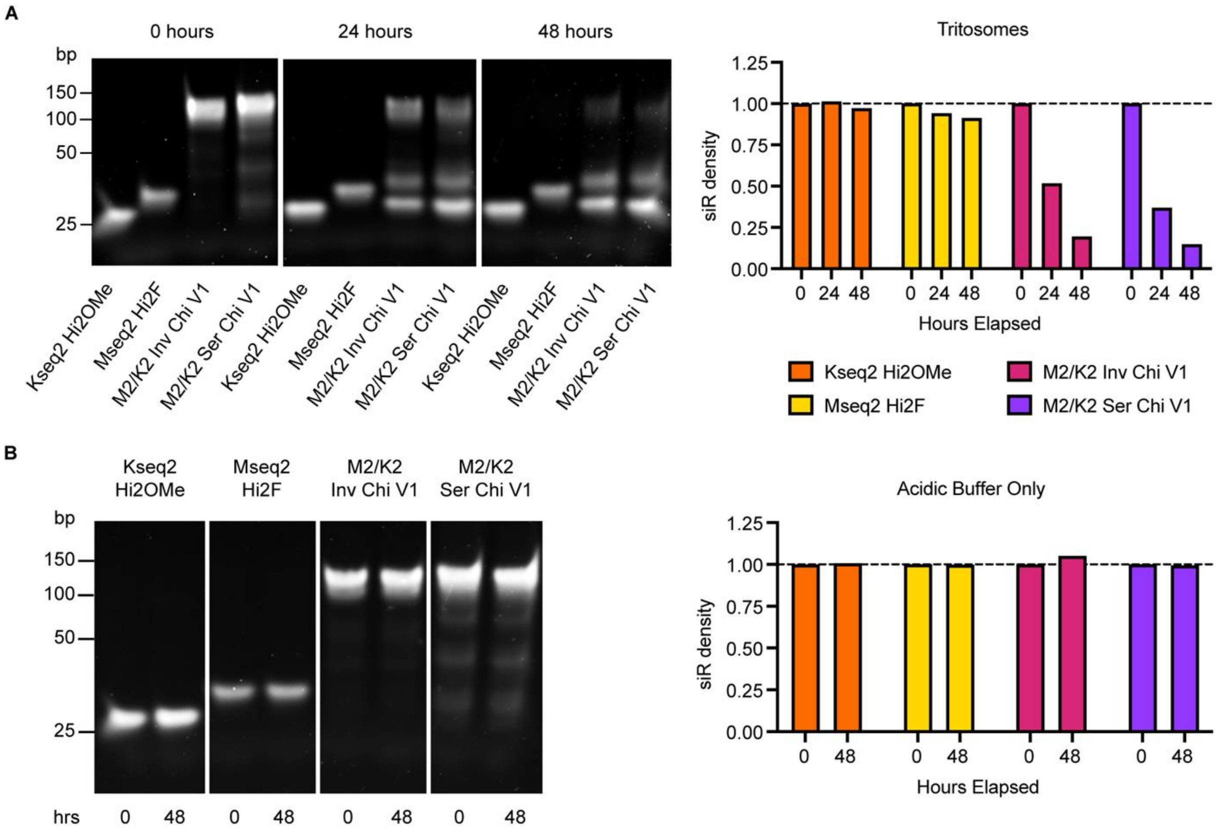

Supplemental Figure 5. Additional assays on MYC-KRAS chimeric siRNA stability. (**A**) Evaluation of siRNA stability in tritosomes. 4 mM of Mseq2 Hi2F, Kseq2 Hi2OMe, M2/K2 Inverted Chimera V1, and M2/K2 Serial Chimera V1 were incubated in acidified rat liver tritosomes for 0, 24, and 48 hrs. (**B**) Evaluation of siRNA stability in acidic buffer. 4 mM of Mseq2 Hi2F, Kseq2 Hi2OMe, M2/K2 Inverted Chimera V1, and M2/K2 Serial Chimera V1 were incubated in 20 mM sodium citrate, pH 5.0 for 0 and 48 hrs. All digestion samples were resolved with 16% acrylamide gel electrophoresis and band intensities were quantified with Image Lab. Relative band intensities were normalized to the 0 hr timepoint for each siRNA.

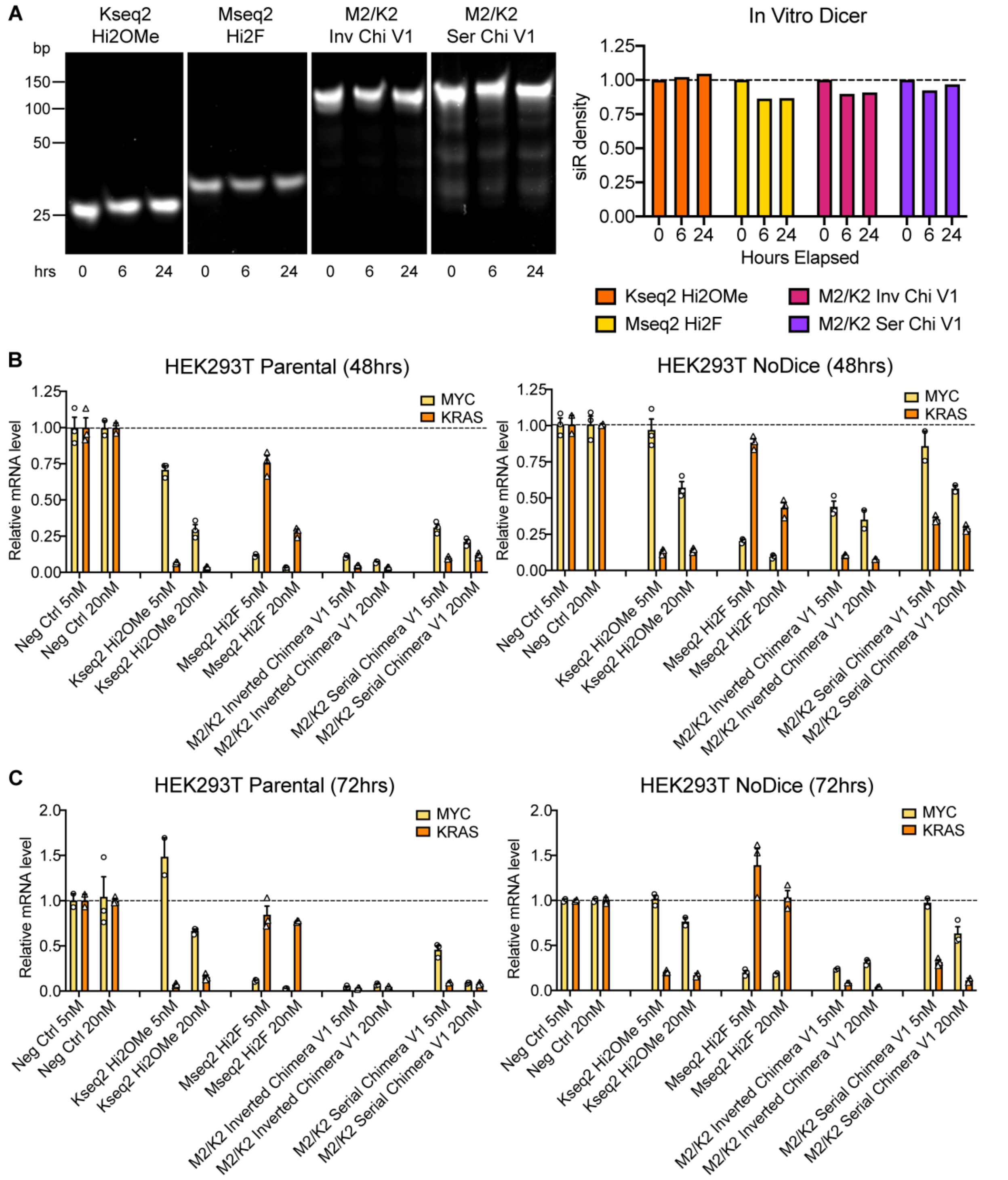

Supplemental Figure 6. Testing DICER activity on MYC/KRAS chimeric siRNA. (**A**) Recombinant Dicer cleavage assay. 10mM of Mseq2 Hi2F, Kseq2 Hi2OMe, M2/K2 Inverted Chimera V1, and M2/K2 Serial Chimera V1 were treated with recombinant human Dicer in buffer

for 0, 6, and 24 hrs. (**B-C**) Relative *MYC* and *KRAS* expression by RT-qPCR in HEK293T parental and NoDice cells following treatment with the negative control siRNA, Mseq2 Hi2F, Kseq2 Hi2OMe, M2/K2 Inverted Chimera V1, and M2/K2 Serial Chimera V1 at 5 and 20nM for (**B**) 48 hrs and (**C**) 72 hrs. Error bars represent SEM.

A

B

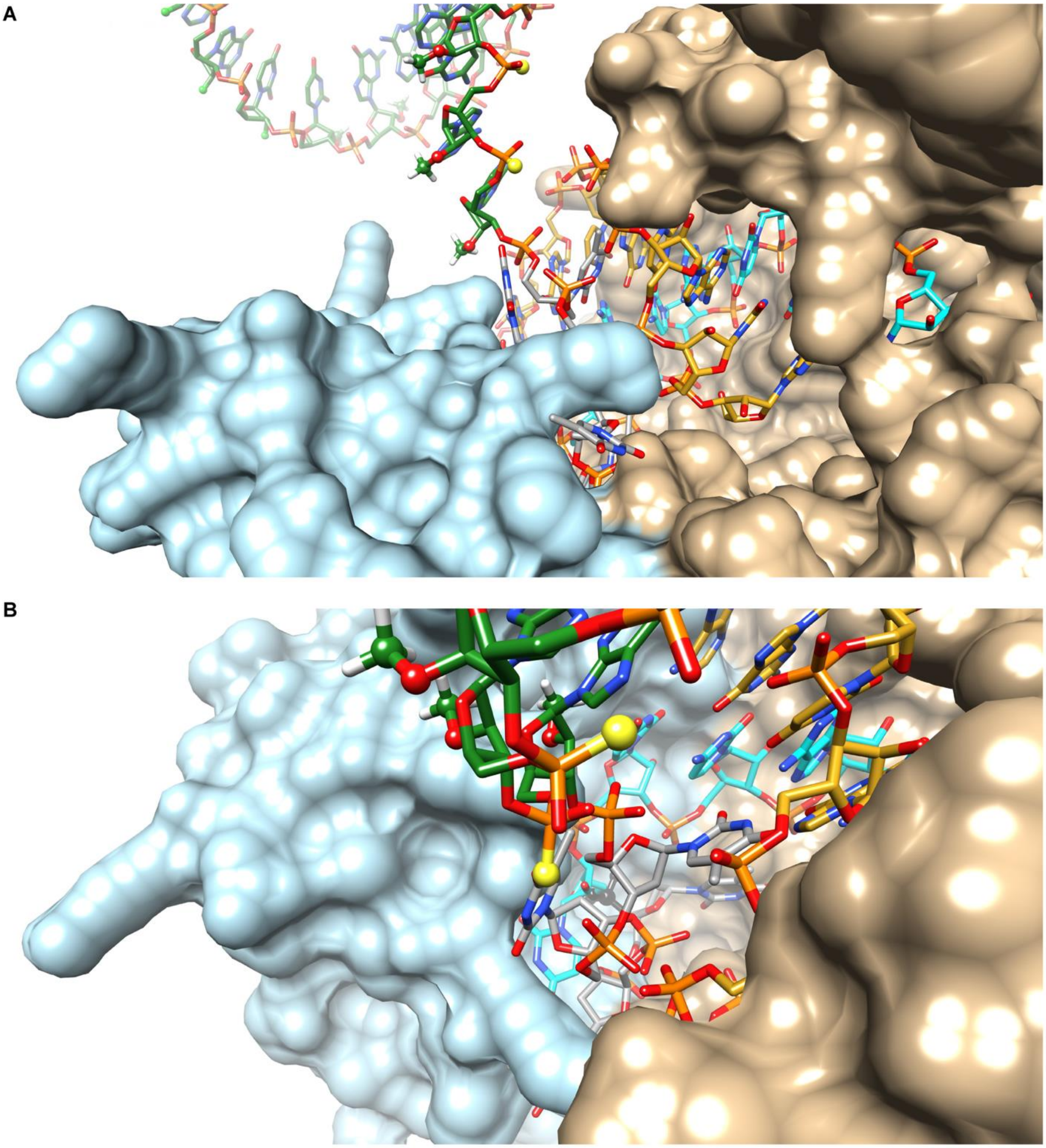

Supplemental Figure 7. Model of inverted chimeric siRNA with uncleaved d(T)$_4$ bridge within Ago2 complex. (**A**) Model showing the uncleaved long chimeric strand fitting into the Ago2 complex, with the Ago2 MID domain residues in light blue and PIWI residues in tan. Carbon atoms of the MYC passenger strand are in green, carbon atoms of the KRAS guide strand are in cyan, and the carbon atoms of the strand representing the targeted KRAS mRNA are in

goldenrod. The thymine bridge is in gray. The KRAS guide and mRNA strands bound to Ago2 portion of this model are based on the crystal structure of Ago2 in complex with an AS:S RNA duplex (PDB ID code 4W5T; Schirle et al., 2014). (**B**) The complex shown in panel A viewed after a 90° rotation around the horizontal axis.

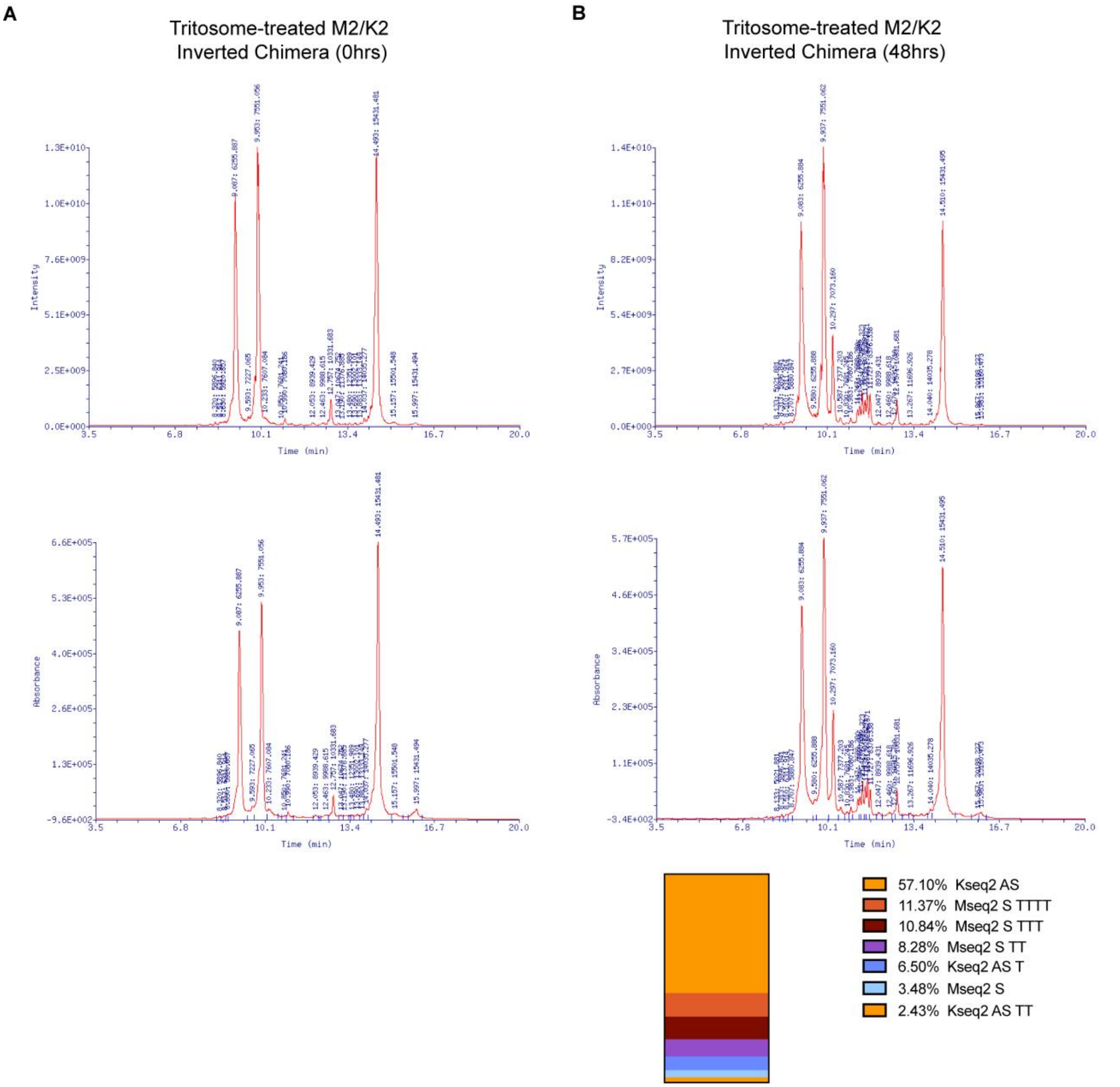

Supplemental Figure 8. LC-MS and LC-UV. (**A**) LC-MS and LC-UV chromatograms for M2/K2 Inverted Chimera V2 treated for 0 hrs with tritosomes. (**B**) LC-MS and LC-UV chromatograms for M2/K2 Inverted Chimera V2 treated for 48 hrs with tritosomes and relative quantification of metabolic cleavage products. Relative values were calculated from the LC/UV peak area percent in Supplemental Table 2. AS = antisense (guide). S = sense (passenger). T = thymidine.

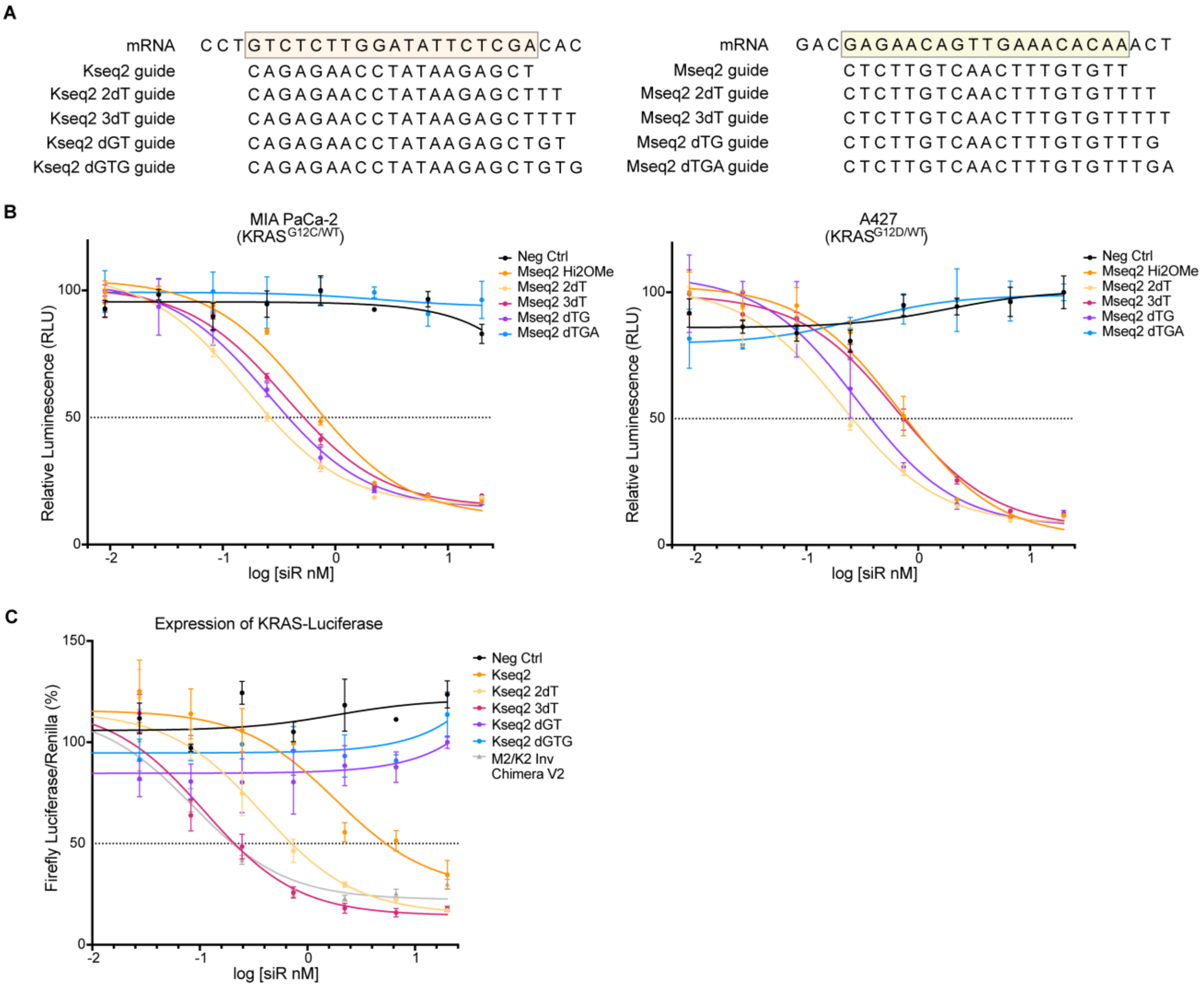

Supplemental Figure 9. Additional assays on characterization of thymidine overhangs. (**A**) Schematic showing the binding sites of Mseq2 and Kseq2 guide strands to their respective target mRNAs and the design of siRNAs with either 2 and 3dT or perfectly complementary overhangs. (**B**) Representative dose-response curves for A427 and MIA-PaCa2 cells treated for six days with the negative control siRNA, Mseq2 Hi2OMe, Mseq2 2dT, Mseq2 3dT, Mseq2 dTG, and Mseq2 dTGA. Error bars represent SEM. (**C**) Representative dose-response curves of KRAS-Firefly luciferase expression in A-431 KRAS-knockout cells treated with the negative control siRNA, Kseq2 Hi2OMe, Kseq2 2dT, Kseq2 3dT, Kseq2 dGT, Kseq2 dGTG, and M2/K2 Inverted Chimera V2. All Firefly-luciferase luminescence values were normalized with Renilla-luciferase luminescence and expressed as a percentage. Error bars represent SEM.

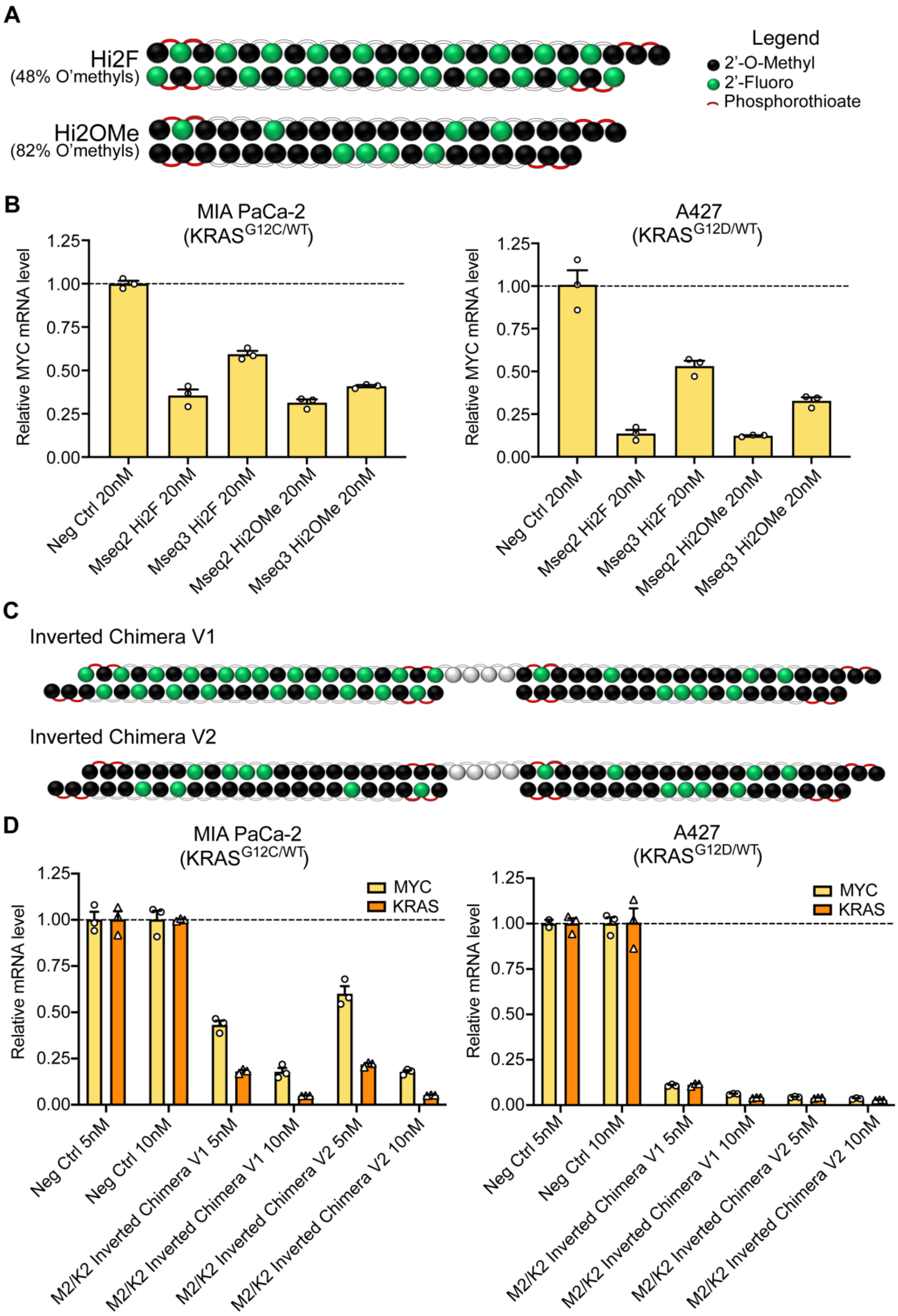
A
Hi2F
(48% O'methyls)
Hi2OMe
(82% O'methyls)
Legend
2'-O-Methyl
2'-Fluoro
Phosphorothioate
B
MIA PaCa-2
(KRAS G12C/WT)
A427
(KRAS G12D/WT)
Relative MYC mRNA level
Neg Ctrl 20nM
Mseq2 Hi2F 20nM
Mseq3 Hi2F 20nM
Mseq2 Hi2OMe 20nM
Mseq3 Hi2OMe 20nM
C
Inverted Chimera V1
Inverted Chimera V2
D
MIA PaCa-2
(KRAS G12C/WT)
A427
(KRAS G12D/WT)
Relative mRNA level
MYC
KRAS
Neg Ctrl 5nM
Neg Ctrl 10nM
M2/K2 Inverted Chimera V1 5nM
M2/K2 Inverted Chimera V1 10nM
M2/K2 Inverted Chimera V2 5nM
M2/K2 Inverted Chimera V2 10nM

Supplemental Figure 10. Optimization of chemical modification pattern on single and chimeric siRNAs. (**A**) Design of Hi2F vs Hi2OMe chemical modification patterns. Hi2F siRNAs contain 48% 2'OMe's whereas Hi2OMe siRNAs contains 82% 2'OMe's. (**B**) Relative *MYC* expression by RT-qPCR in MIA PaCa-2 and A427 cells following treatment with the negative control, two MYC Hi2F, and two MYC Hi2OMe siRNAs at 20nM for 72 hrs. Error bars represent SEM. (**C**) Design of V1 and V2 inverted chimeric siRNAs. The V2 Inverted Chimera is fully Hi2OMe-modified on both the KRAS and MYC siRNAs. (**D**) Relative *MYC* and *KRAS* expression by RT-qPCR in MIA PaCa-2 and A427 cells following treatment with the negative control siRNA, MYC Hi2OMe, KRAS Hi2OMe, M2/K2 Inverted Chimera V1, and M2/K2 Inverted Chimera V2 at 5 and 10nM for 72 hrs. Error bars represent SEM.

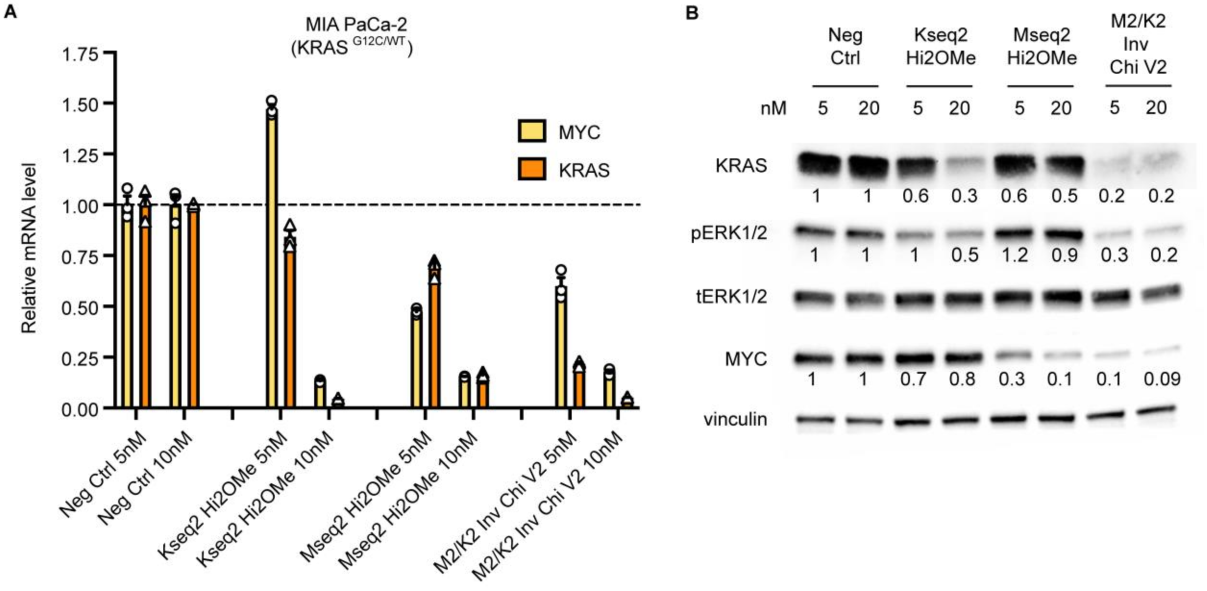

Supplemental Figure 11. Additional assays on activity of the fully Hi2OMe-modified M2/K2 inverted chimeric siRNA. (**A**) Relative *MYC* and *KRAS* expression by RT-qPCR in MIA PaCa-2 cells following treatment with the negative control siRNA, MYC Hi2OMe, KRAS Hi2OMe, and M2/K2 Inverted Chimera V2 at 5 and 10nM for 72 hrs. Error bars represent SEM. (**B**) KRAS, phospho-ERK1/2, total ERK1/2, and MYC expression by Western blot in MIA PaCa-2 cells following treatment with the control siRNA, MYC Hi2OMe, KRAS Hi2OMe, and M2/K2 Inverted Chimera V2 at 5 and 20nM for 72 hrs. Band intensities were quantified with Image Lab. Relative band intensities were calculated in comparison to control siRNA-treated cells at the comparative dose and expression of each protein were normalized as follows: KRAS, phospho-S6, and MYC were normalized to vinculin, and phospho-ERK1/2 was normalized to vinculin and then total-ERK1/2. Relative expression values are shown below each band for KRAS, phospho-ERK1/2, and MYC.

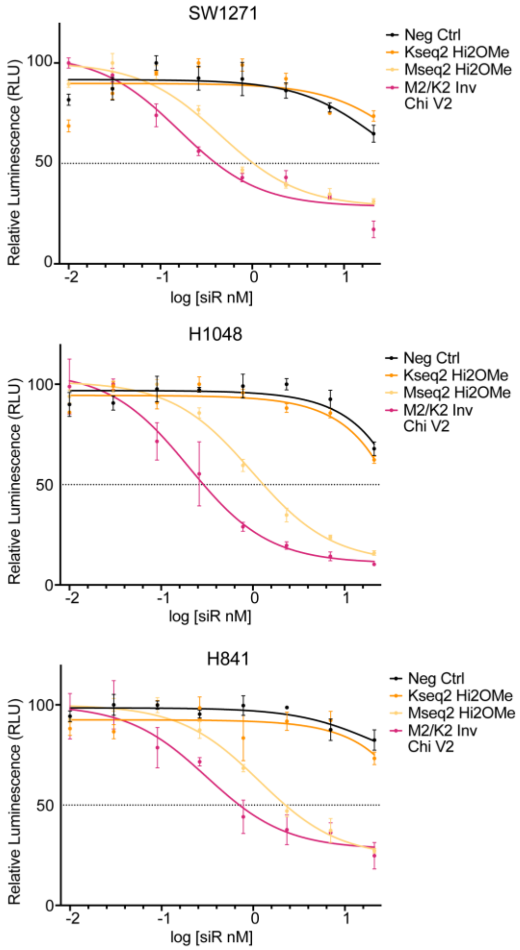

Supplemental Figure 12. In vitro activity of MYC/KRAS chimeric siRNA on MYC-dependent small cell lung cancer lines. Representative dose-response curves for SW 1271, H1048, and H841 cells treated for six days with the negative control siRNA, Mseq2 Hi2OMe, Kseq2 Hi2OMe, and M2/K2 Inverted Chimera V2. Error bars represent SEM.

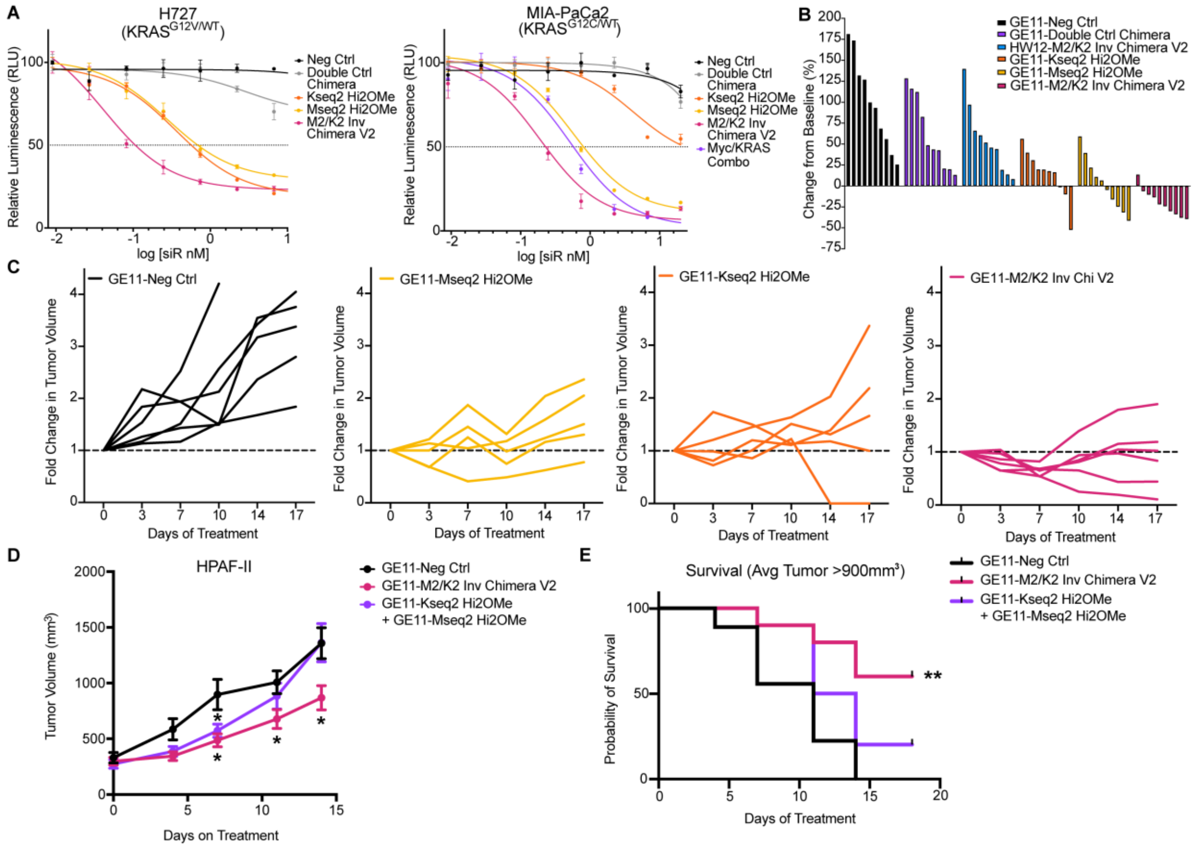

Supplemental Figure 13. Additional assays on in vivo activity and efficacy of M2/K2 inverted chimeric siRNA. (**A**) Representative dose-response curves for H727 and MIA-PaCa2 cells treated for six days with the negative single siRNA control, double control siRNA, Mseq2 Hi2OMe, Kseq2 Hi2OMe, M2/K2 Inverted Chimera V2 and Mseq2 + Kseq2 siRNA co-transfection. In conditions with MYC + KRAS co-transfection, each of the MYC and KRAS siRNAs were transfected at the indicated dose. Error bars represent SEM. (**B**) Percent change in H727 tumor volume for each mouse from baseline after eight days of siRNA treatment. (**C**) Spider plots of fold changes in A427 tumor volume for every mouse in each treatment group over 21 days. (**D**) Tumor growth curves showing change in average HPAF-II tumor volume over 14 days (n=9-10 for all treatment groups). Error bars represent SEM. Unpaired one-tailed t-test corrected for multiple comparisons using the Bonferroni method was used for statistical comparisons. (*) = $p<0.05$. (**E**) Survival plot for HPAF-II model of average tumor size >900mm^3 over 18 days. Log-rank (Mantel-Cox) test was used to calculate statistical significance. (**) = $p<0.01$.

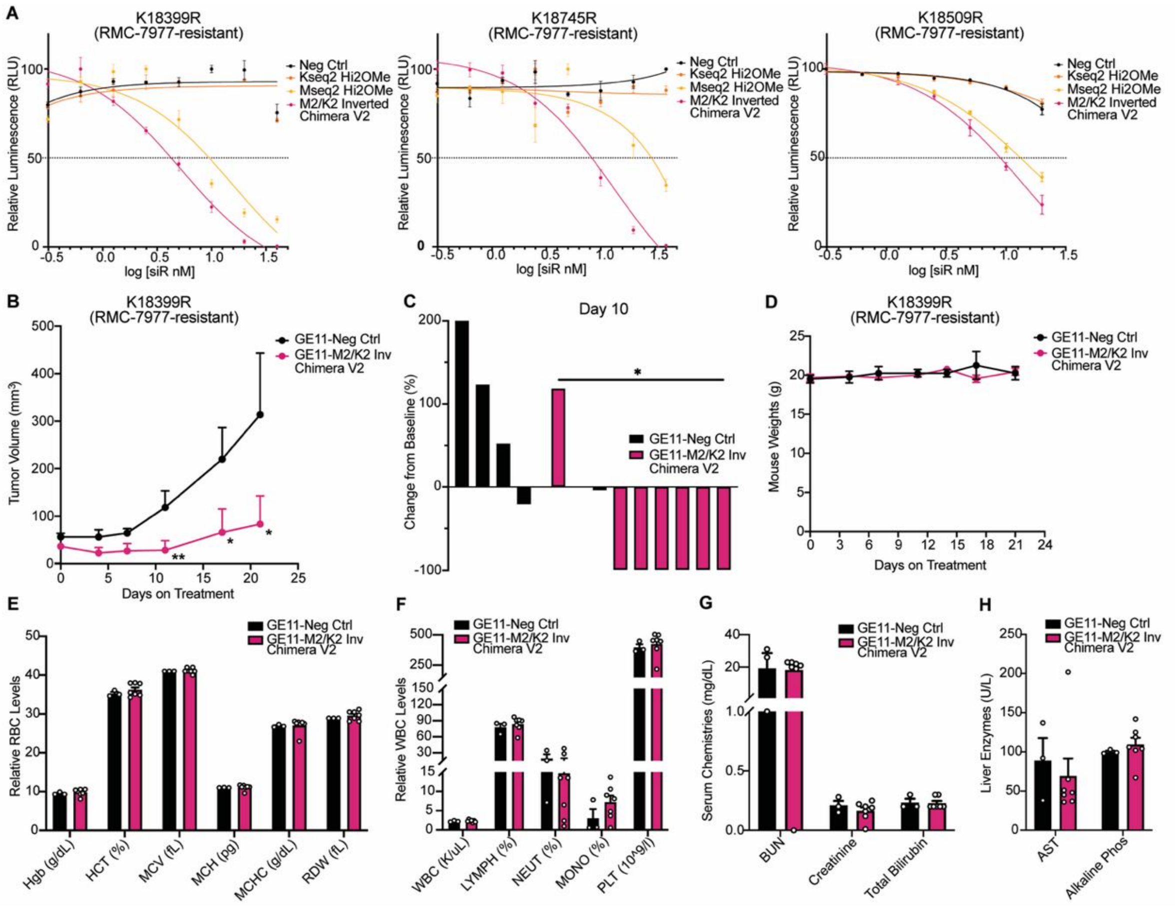

Supplemental Figure 14. In vivo activity and efficacy of M2/K2 inverted chimeric siRNA in a murine KRAS inhibitor-resistant model. (**A**) Representative dose-response curves for K18399R, K18745R, and K18509R cells treated for six days with the negative single siRNA control, Mseq2 Hi2OMe, Kseq2 Hi2OMe, and M2/K2 Inverted Chimera V2. Error bars represent SEM. (**B**) Tumor growth curves showing change in average K18399R tumor volume over 21 days (n=4-9 for all treatment groups). Error bars represent SEM. Unpaired Mann-Whitney test was used for statistical comparisons. (**) = p<0.01, (*) = p<0.05. (**C**) Percent change in K18399R tumor volume for each mouse from baseline after ten days of siRNA treatment. Fisher's exact test was used for statistical significance. (*) = p<0.05. (**D**) Average mouse weights over 21 days of siRNA treatment. Blood studies for (**E**) red blood cell counts, (**F**) complete blood cell differentials, (**G**) kidney and liver function, and (**H**) liver enzymes were obtained at the end of the study.

A

p-values:	Day 7	Day 11	Day 14	Day 18
GE11-Kseq2 Hi2OMe	ns	ns	ns	*
GE11-Mseq2 Hi2OMe	ns	ns	ns	ns
GE11-M2/K2 Inv Chimera	*	*	*	**
AMG510	ns	**	**	**
AMG510 + GE11-M2/K2 Inv Chimera	**	***	***	***

B

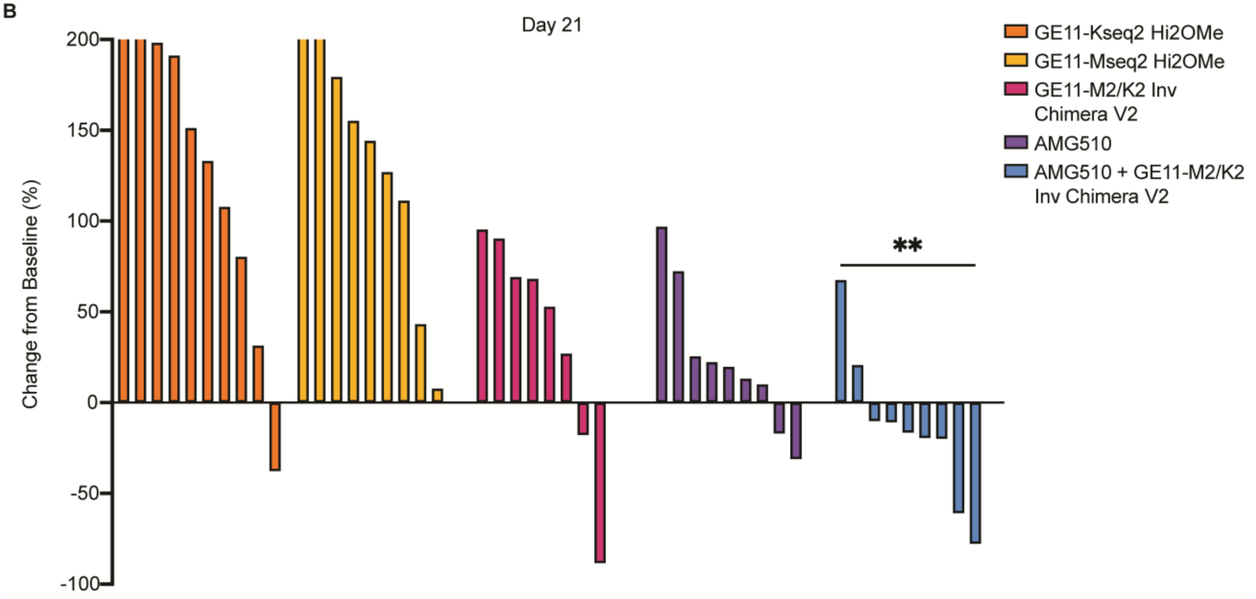

Supplemental Figure 15. Additional assays on long-term in vivo efficacy of M2/K2 inverted chimeric siRNA. (**A**) Table with p-values of each group compared to tumor volumes in the negative control-treated group. Values are based on unpaired one-tailed t-tests corrected for multiple comparisons using the Bonferroni method. (**B**) Percent change in H358 tumor volume for each mouse from baseline after twenty-one days of siRNA treatment. Fisher's exact test was used for statistical comparisons with the negative control-treated group (not shown). (***) = p<0.001, (**) = p<0.01, = p (*) = p<0.05.

Supplemental Table 1. siRNA sequences.

Supplemental Table 2. LC-MS chromatogram summary.

Supplemental Table 3. Primer sequences for real-time quantitative PCR.

References

1. Bogerd HP, Whisnant AW, Kennedy EM, Flores O, and Cullen BR. Derivation and characterization of Dicer- and microRNA-deficient human cells. *RNA.* 2014;20(6):923-37.
2. Wasko UN, Jiang J, Dalton TC, Curiel-Garcia A, Edwards AC, Wang Y, et al. Tumour-selective activity of RAS-GTP inhibition in pancreatic cancer. *Nature.* 2024;629(8013):927-36.
3. Papke B, Azam SH, Feng AY, Gutierrez-Ford C, Huggins H, Pallan PS, et al. Silencing of Oncogenic KRAS by Mutant-Selective Small Interfering RNA. *ACS Pharmacol Transl Sci.* 2021;4(2):703-12.
4. Zheng G, Lu XJ, and Olson WK. Web 3DNA--a web server for the analysis, reconstruction, and visualization of three-dimensional nucleic-acid structures. *Nucleic Acids Res.* 2009;37(Web Server issue):W240-6.
5. Pettersen EF, Goddard TD, Huang CC, Couch GS, Greenblatt DM, Meng EC, et al. UCSF Chimera--a visualization system for exploratory research and analysis. *J Comput Chem.* 2004;25(13):1605-12.
6. Schirle NT, Sheu-Gruttadauria J, and MacRae IJ. Structural basis for microRNA targeting. *Science.* 2014;346(6209):608-13.
7. Case DA, Cheatham TE, 3rd, Darden T, Gohlke H, Luo R, Merz KM, Jr., et al. The Amber biomolecular simulation programs. *J Comput Chem.* 2005;26(16):1668-88.
8. Illumina. https://www.illumina.com/systems/sequencing-platforms/novaseq.html. 2023.
9. Dobin A, Davis CA, Schlesinger F, Drenkow J, Zaleski C, Jha S, et al. STAR: ultrafast universal RNA-seq aligner. *Bioinformatics.* 2013;29(1):15-21.
10. Patro R, Duggal G, Love MI, Irizarry RA, and Kingsford C. Salmon provides fast and bias-aware quantification of transcript expression. *Nat Methods.* 2017;14(4):417-9.
11. Frankish A, Diekhans M, Ferreira AM, Johnson R, Jungreis I, Loveland J, et al. GENCODE reference annotation for the human and mouse genomes. *Nucleic Acids Res.* 2019;47(D1):D766-D73.
12. Love MI, Huber W, and Anders S. Moderated estimation of fold change and dispersion for RNA-seq data with DESeq2. *Genome Biol.* 2014;15(12):550.
13. Zhu A, Ibrahim JG, and Love MI. Heavy-tailed prior distributions for sequence count data: removing the noise and preserving large differences. *Bioinformatics.* 2019;35(12):2084-92.
14. International HGOH-H. https://www.hugo-international.org/.
