# Supplementary figures and images for: Inverted chimeric RNAi molecules synergistically cotarget MYC and KRAS in KRAS-driven cancers
Source: J Clin Invest. 2025 Jul 22;135(19):e187204. doi: 10.1172/JCI187204 (PMC12483557; doi:10.1172/JCI187204)

Fig 2a

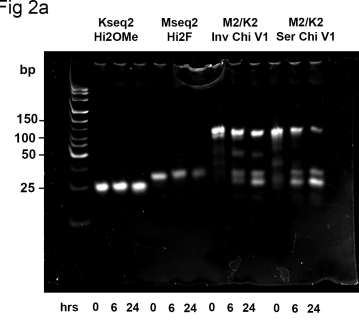

Fig 2b

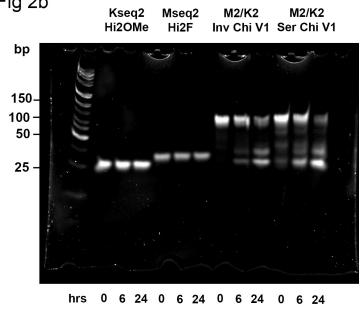

Fig 2c

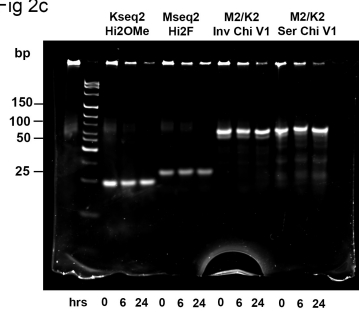

Fig 5d

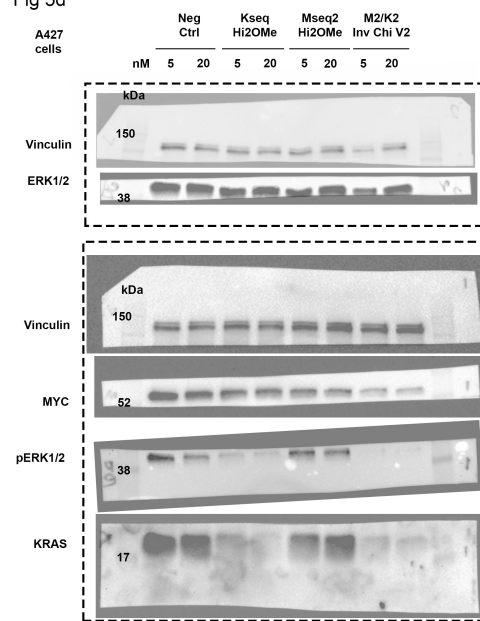

Fig 8e

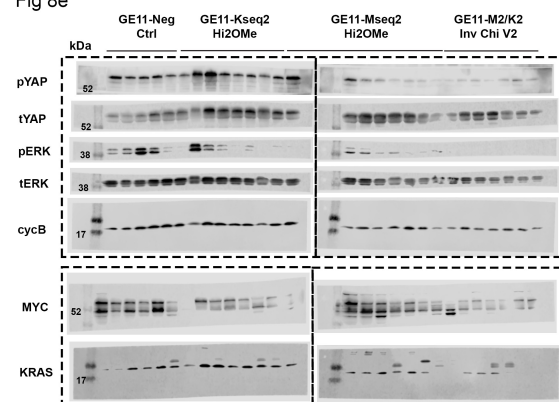

Supp Fig 1c

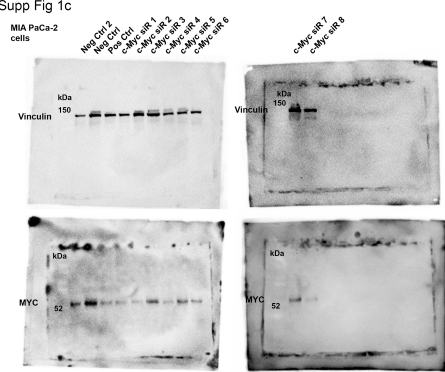

Supp Fig 2b

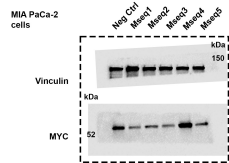

Supp Fig 4c

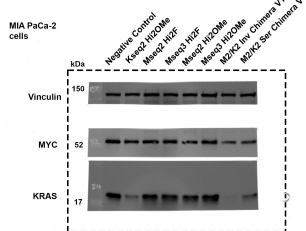

Supp Fig 5a

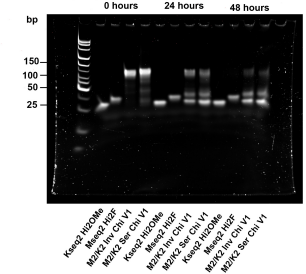

Supp Fig 5b

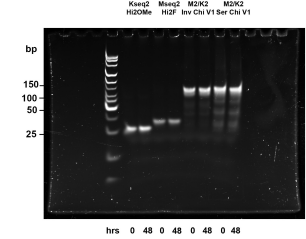

Supp Fig 6a

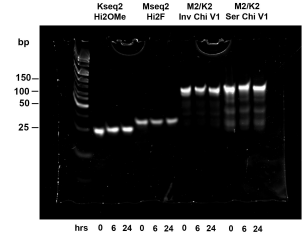

Supp Fig 11b

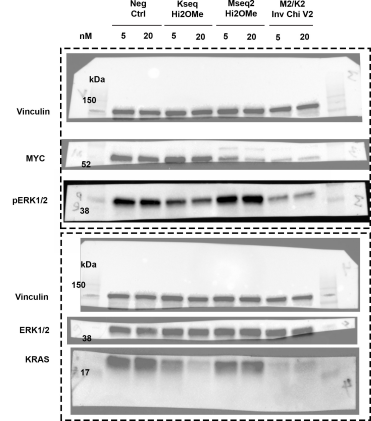

Supplement: Unedited blot and gel images [file jci-135-187204-s163.pdf]
